# MicroRNA profile for health risk assessment: Environmental exposure to persistent organic pollutants strongly affects the human blood microRNA machinery

**DOI:** 10.1038/s41598-017-10167-7

**Published:** 2017-08-23

**Authors:** Julian Krauskopf, Theo M. de Kok, Dennie G. Hebels, Ingvar A. Bergdahl, Anders Johansson, Florentin Spaeth, Hannu Kiviranta, Panu Rantakokko, Soterios A. Kyrtopoulos, Jos C. Kleinjans

**Affiliations:** 1 Department of Toxicogenomics, Maastricht University, Maastricht, The Netherlands; 2 Department of Biobank Research, and Occupational and Environmental Medicine, Department of Public Health and Clinical Medicine, Umeå University, Umeå, Sweden; 3 Nutrition Research, Department of Public Health and Clinical Medicine, Umeå University, Umeå, Sweden; 4 Faculty of Medicine, Department of Radiation Sciences, Oncology, Umeå University, Umeå, Sweden; 5 Department of Health Protection, Chemicals and Health Unit, National Institute for Health and Welfare, Kuopio, Finland; 6 Institute of Biology, Medicinal Chemistry and Biotechnology, National Hellenic Research Foundation, Athens, Greece

## Abstract

Persistent organic pollutants (POPs) are synthetic chemical substances that accumulate in our environment. POPs such as polychlorinated biphenyls (PCBs), hexachlorobenzene (HCB) and dichlorodiphenyltrichloroethane (DDT) have been classified as carcinogenic to humans and animals. Due to their resistance to biodegradation humans are still exposed to these compounds worldwide. We aim to evaluate the miRNA and transcriptomic response of a human population exposed to POPs. The miRNA and transcriptomic response was measured in blood of healthy subjects by microarray technology and associated with the serum concentrations of six PCB congeners, DDE (a common DDT metabolite), and HCB. A total of 93 miRNA levels appeared significantly associated with the POP-exposure (FDR < 0.05). The miRNA profile includes four tumor suppressor miRNAs, namely miR-193a-3p, miR-152, miR-31-5p and miR-34a-5p. Integration of the miRNA profile with the transcriptome profile suggests an interaction with oncogenes such as *MYC*, *CCND1*, *BCL2* and *VEGFA*. We have shown that exposure to POPs is associated with human miRNA and transcriptomic responses. The identified miRNAs and target genes are related to various types of cancer and involved in relevant signaling pathways like wnt and p53. Therefore, these miRNAs may have great potential to contribute to biomarker-based environmental health risk assessment.

## Introduction

Persistent organic pollutants (POPs) are synthetic chemical substances that persist in the environment and accumulate in high concentrations in fatty tissues. Throughout the 20^th^ century these chemicals were widely used as pesticides, such as hexachlorobenzene (HCB) and dichlorodiphenyltrichloroethane (DDT), or as industrial chemicals as is the case for polychlorinated biphenyls (PCBs). Because of their resistance to biodegradation and environmental toxicity, these compounds have been banned (HCB and PCBs), or restricted (DDT) by the Stockholm Convention on Persistent Organic Pollutants in 2001^1^. However, due to earlier use and biomagnification of POPs in the food chain, humans are still exposed worldwide mainly as a consequence of dietary intake, and of exposure to air and water pollution^2^. This is of a major concern as PCBs have been classified as definite, HCB as probable and DDT as possible human carcinogens by the International Agency of Research on Cancer^3^. Several epidemiological studies suggest that PCB, but also organochlorine pesticides such as DDT and HCB levels measured in peripheral blood, are related to increased risk of multiple types of cancer, including non-Hodgkin lymphoma and breast cancer^4–6^.

Several omics studies have been conducted to better understand the relationship between cancer risks and environmental exposure by investigating the transcriptomics, metabolomics and proteomics responses to exposure to carcinogenic compounds, predominantly in peripheral blood samples. Recently, it has been shown that also the human microRNA (miRNA) machinery is altered in response to environmental carcinogens, interestingly before the onset of cancer^7^. These small non-coding RNA sequences (~22 nucleotides), regulating gene expression at the posttranscriptional level, are involved in all fundamental processes such as development, growth, differentiation, immune reaction, and adaptation to stress^8^. This large regulatory potential widely impacts the development and progression of cancer upon exposure-induced modulation of gene expression^9^. Therefore, these key regulators of disease related molecular mechanisms may have great potential as novel biomarkers of exposure and cancer risks^10^.

In this study we establish the impact of environmental carcinogens on miRNA and transcriptomic profiles in buffy coats of healthy subjects drawn from the general population. The present study was conducted in the context of the EnviroGenomarkers project^11^. The samples were selected from –at that time- healthy subjects of the Northern Sweden Health and Disease Study (NSHDS) some of whom eventually developed lymphoma. For the present study we generated the miRNA and transcriptomic expression profiles from buffy coats. The exposure markers of POPs were measured in serum^12^. We applied a linear model to relate a combination of 6 PCB congeners, HCB and DDE (a breakdown product of DDT) to the miRNA profile which was subsequently integrated with the transcriptome profile. To the best of our knowledge this is the first report on a population-based study showing that the miRNA machinery acts in concert with the transcriptome upon exposure to a combination of environmental carcinogens.

## Results

**Figure 1:**
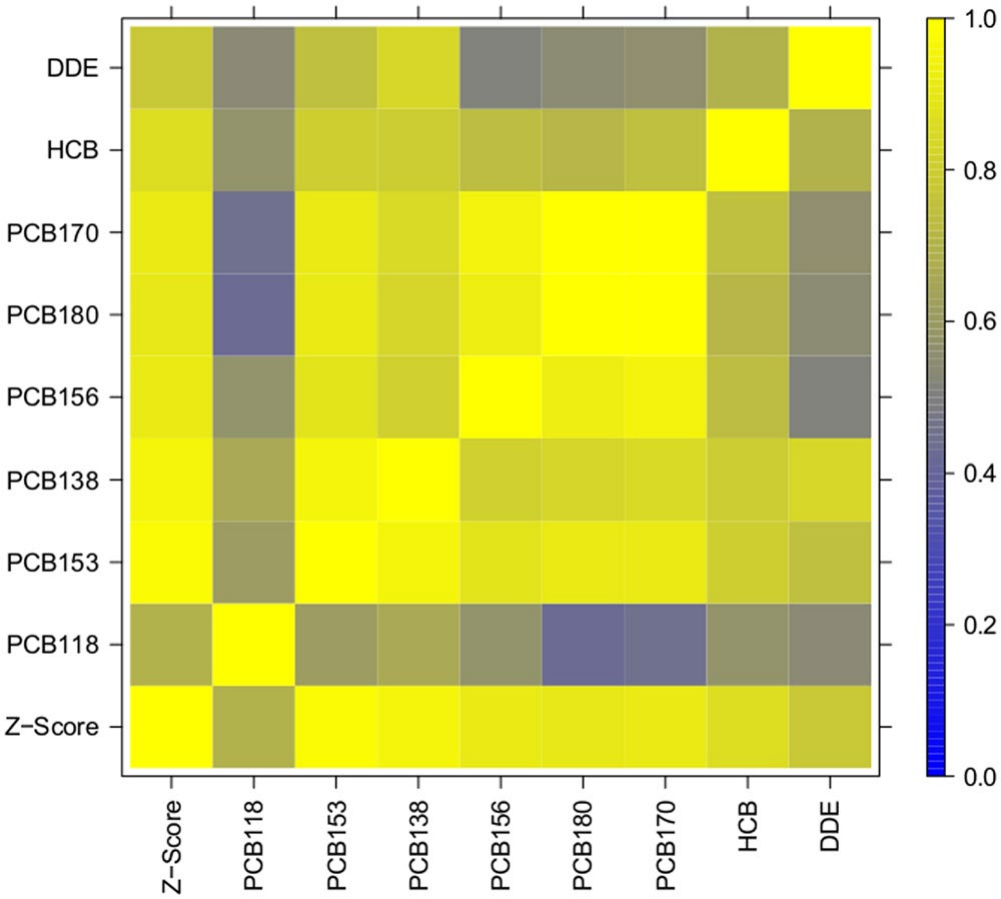
Pearson correlation among the eight POPs and the Z-Score. The individual levels of PCB153, PCB138, PCB156, PCB170, PCB180, HCB and DDE highly correlated with the cumulative Z-Score (r between 0.81 and 0.98). Only PCB118 showed a marginally lower correlation (r = 0.77).

We examined the impact of 6 PCB congeners, HCB and DDE on the blood miRNA machinery of a total of 207 subjects. The individual levels of PCB153, PCB138, PCB156, PCB170, PCB180, HCB and DDE^13^ highly correlated with the cumulative Z-Score (correlation coefficient (r) between 0.81 and 0.98) (Fig. 1). Only PCB118, a congener that in contrast to the other congeners has a coplanar structure, showed a marginally lower correlation (r = 0.77). The median, mean and range of the individual POP exposure and the cumulative Z-Score for the selected individuals is shown in Table 1.

**Table 1:** Median, mean and range of the exposure to the individual POPS and the cumulative Z-Score.

	Median	Mean	Range
PCB118	105.32	142.62	8.45, 832.08
PCB153	968.81	1127.91	32.47, 4334.12
PCB138	522.07	605.18	10.97, 2675.35
PCB156	88.89	102.76	15.5, 394.89
PCB180	664.73	744.6	100.08, 2159.5
PCB170	336.3	378.07	50.39, 1211.85
HCB	202.51	229.84	64.52,706.61
DDE	1425.06	2228.92	16.4, 18041.58
Z-Score	−1.82	0	−11.05, 38.03

### Regulation of miRNA expression by POPs

**Figure 2:**
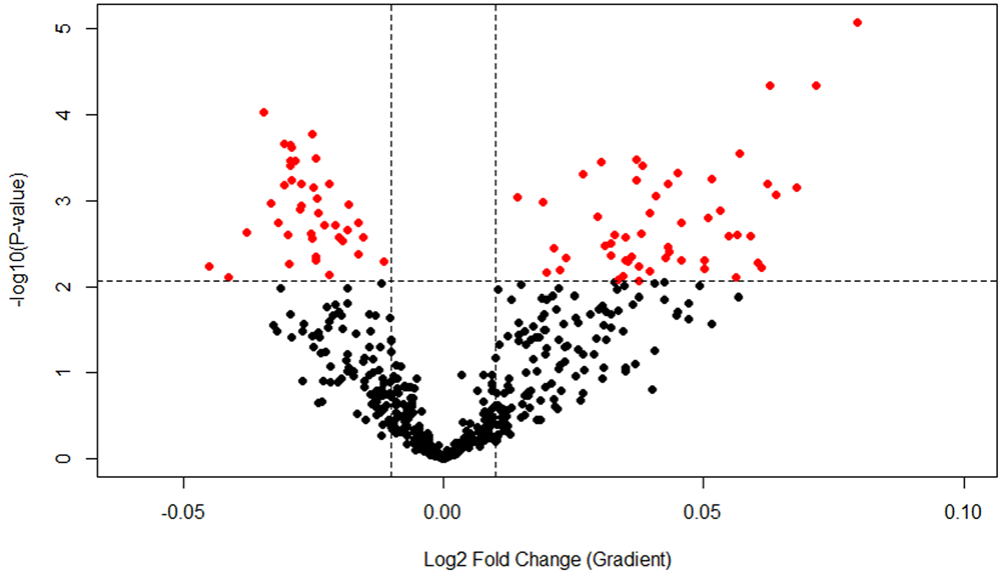
Volcano plot representing all identified miRNAs. For each miRNA identified in this study the volcano plot shows the fold change (gradient of the association with the cumulative Z-Score) against the –log *P* value. Statistically significant associated miRNAs are depicted as red dots.

**Figure 3:**
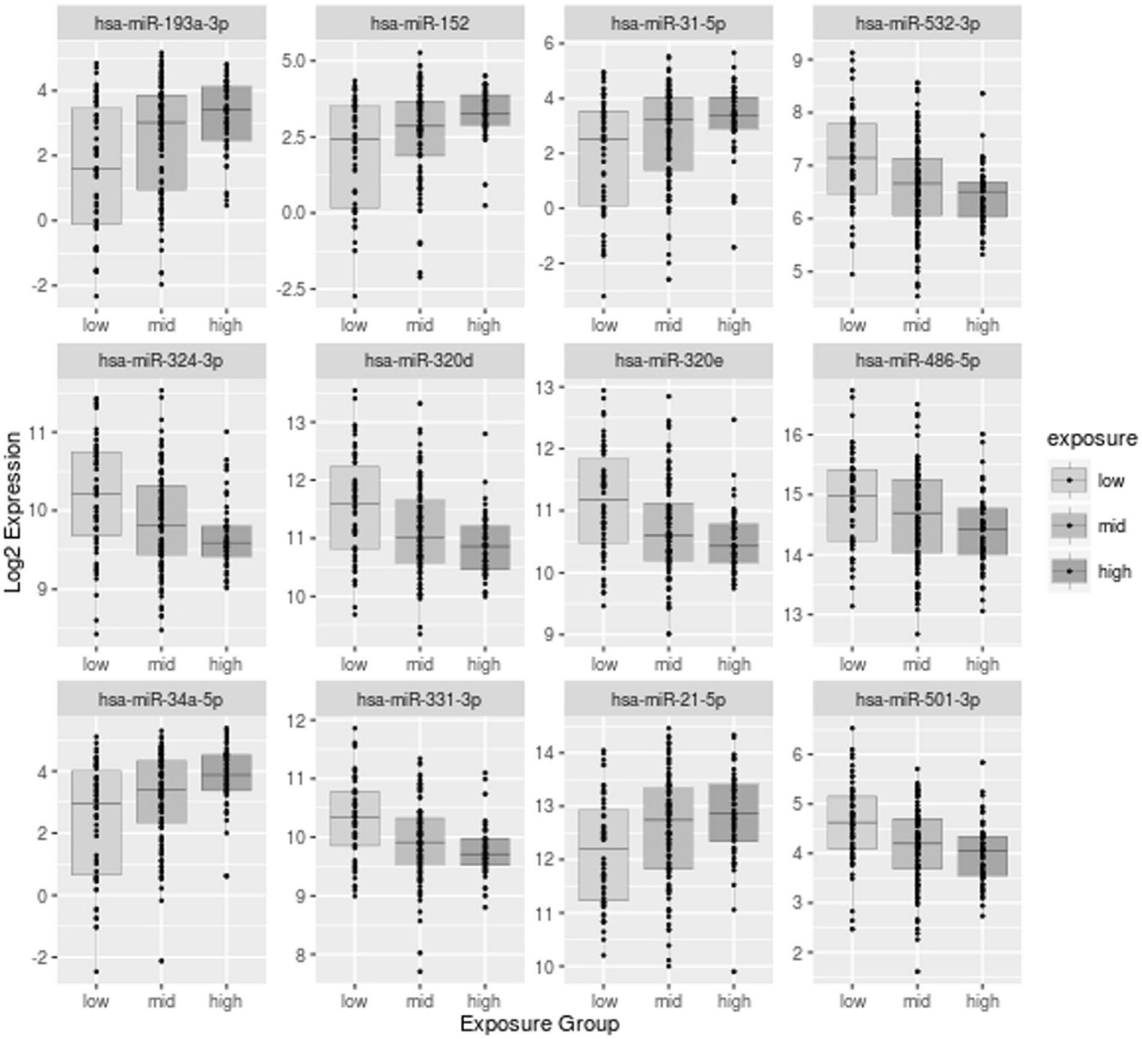
Expression levels of the top 12 associated miRNAs in the defined exposure groups. To visualize the effect of exposure on the miRNAs we divided the cohort, based on the quantiles of the cumulative Z-Score, into low, middle and high exposed subjects (54, 100 and 53 subjects; 1^st^, 2^nd^ + 3^rd^, 4^th^ quantile respectively).

The linear model identified 93 out of the 543 observed miRNA expressions to be significantly associated with the cumulative Z-Score of POP exposure (FDR < 0.05). Of these miRNAs 53 were positively and 40 negatively correlated with the cumulative Z-Score (Fig. 2, Supplementary Table S1). To visualize the effect of the POP exposure on the miRNA expression we divided the cohort, based on the quantiles of the Z-Score of POPs’, into low, middle and high exposed subjects (54, 100 and 53 subjects; 1^st^, 2^nd^ + 3^rd^, 4^th^ quantile respectively). Figure 3 shows the exposure-related intensity of the top 12 significantly associated miRNAs (ranked by FDR).

We also applied the linear model to the individual POPs and observed for PCB138 84 associations, PCB153 121 associations, PCB156 32 associations, PCB170 90 associations, PCB180 82 associations and for HCB 67 associations. For PCB118 and DDE, we did not find any associations with a FDR < 0.05. The signature of the Z-Score of POPs included between 70 and 100 percent of the individual POP associations (Supplementary Table S2). Furthermore, we applied the linear model of the cumulative Z-Score also to the stratified data for only males, females, smokers, non-smokers, future lymphoma cases and controls; however, after applying the correction for multiple testing only 32 associations were found for the subset of the non-smokers and 36 associations among the healthy controls. For only males, females, smokers and future cases no association had a FDR < 0.05 (Supplementary Table S3).

### Associations between miRNAs and the transcriptome

The experimentally validated microRNA-target interactions database miRTarBase found a total of 7175 target genes for the identified set of miRNAs. Upon calculating the Pearson-correlation of the identified miRNAs with the previously obtained transcriptomics data^14^ we identified 217 target genes to be inversely correlated with their respective miRNA (r < −0.4 and FDR < 0.05). Using the gene set analysis of ConsensusPathDB we found that 20 KEGG pathways, including pathways in a range of human cancers, were associated with the list of inversely correlated target genes (q-value < 0.05) (Table 2). Furthermore, we observed an overrepresentation of 148 GO terms in the category of biological processes such as Wnt signaling pathway, apoptotic process, and regulation of cell cycle process (Supplementary Table S4).

**Table 2:** Associated KEGG pathways from inversely correlated target gene expressions. Cancer related pathways are marked in bold.

KEGG Pathway	Size	Targets	Targets contained	q-value
Focal adhesion - Homo sapiens (human)	207	11	5.30%	0.000422
Ribosome - Homo sapiens (human)	137	9	6.70%	0.000422
**Proteoglycans in cancer - Homo sapiens (human)**	204	10	4.90%	0.00116
**Pathways in cancer - Homo sapiens (human)**	398	14	3.50%	0.00116
**Wnt signaling pathway - Homo sapiens (human)**	140	8	5.80%	0.00155
Hippo signaling pathway - Homo sapiens (human)	154	8	5.20%	0.00251
**PI3K-Akt signaling pathway - Homo sapiens (human)**	347	12	3.50%	0.00268
**Small cell lung cancer - Homo sapiens (human)**	86	6	7.00%	0.00272
**Prostate cancer - Homo sapiens (human)**	89	6	6.70%	0.00292
**Bladder cancer - Homo sapiens (human)**	41	4	9.80%	0.0071
Adherens junction - Homo sapiens (human)	73	5	6.80%	0.00726
Viral myocarditis - Homo sapiens (human)	58	4	6.90%	0.0214
**Thyroid cancer - Homo sapiens (human)**	29	3	10.30%	0.0214
Signaling pathways regulating pluripotency of stem cells - Homo sapiens (human)	142	6	4.20%	0.0214
**Colorectal cancer - Homo sapiens (human)**	62	4	6.50%	0.0222
HIF-1 signaling pathway - Homo sapiens (human)	103	5	4.90%	0.0229
**Chronic myeloid leukemia - Homo sapiens (human)**	73	4	5.50%	0.035
Thyroid hormone signaling pathway - Homo sapiens (human)	119	5	4.20%	0.0356
Protein processing in endoplasmic reticulum - Homo sapiens (human)	169	6	3.60%	0.0356
Bacterial invasion of epithelial cells - Homo sapiens (human)	78	4	5.10%	0.0375

We have also observed 253 positive correlations of a miRNA with its target gene (r > 0.4 and FDR < 0.05). For the positively correlated genes we found 6 KEGG pathways, including small cell lung cancer, p53 signaling pathway and chronic myeloid leukemia, to be enriched (q-value < 0.05) (Table 3). Four out of these 6 also appeared among the pathways retrieved from inversely correlated genes. For the positively correlated genes we identified 57 overrepresented GO terms that included biological processes relevant to carcinogenesis (Supplementary Table S5). To validate the associations of the inversely and positively correlated genes with an independent tool, we also performed a gene set analysis using the Molecular Signatures Database^15^. Indeed, we observed significant overlap with the hallmark and oncogenic gene sets from the Molecular Signatures Database (FDR < 0.05) (Supplementary Tables S6 and S7).

**Table 3:** Associated KEGG pathways from positively correlated target gene expressions. Cancer related pathways are marked in bold.

KEGG Pathway	Size	Targets	Targets contained	q-value
**Small cell lung cancer - Homo sapiens (human)**	86	6	7.00%	0.0288
**p53 signaling pathway - Homo sapiens (human)**	68	5	7.40%	0.03
**Chronic myeloid leukemia - Homo sapiens (human)**	73	5	6.80%	0.03
Hippo signaling pathway - Homo sapiens (human)	154	7	4.60%	0.03
Viral myocarditis - Homo sapiens (human)	58	4	6.90%	0.0476
Pentose phosphate pathway - Homo sapiens (human)	29	3	10.30%	0.0476

### Potential interference of miRNAs in human cancer

**Figure 4:**
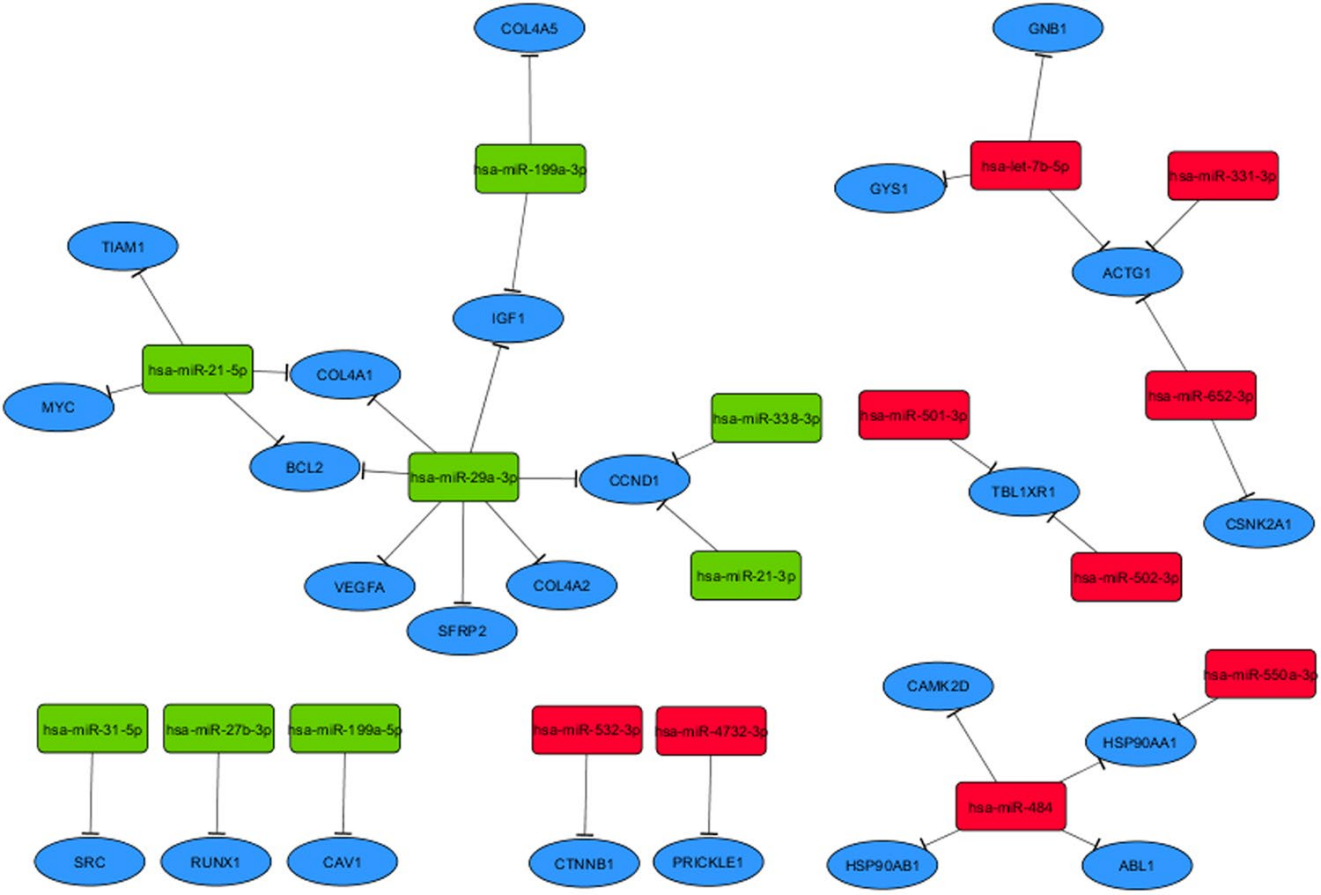
Potential interference of miRNAs with human cancer genes upon environmental exposure to PCBs, HCB and DDE. Red color indicates a downregulation and green color an upregulation of a miRNA with the exposure intensity. Blue color indicates cancer related genes derived from the KEGG pathways. All interactions are inverse correlations (r < −0.4 and FDR < 0.05).

Figure 4 presents the potential interference of the POP exposure-associated miRNAs with the inversely regulated gene targets that were derived from the cancer-related KEGG pathways (Table 2). The eight upregulated miRNAs (green) target 13 inversely regulated genes associated with cancer. The miRNA miR-29a plays a major role in this network as it inversely correlates with eight cancer-related gene targets. Furthermore, we detected nine downregulated miRNAs (red) inversely correlating with 11 cancer-associated genes (Fig. 4).

## Discussion

In this study we investigated the impact of exposure to carcinogenic POPs on the miRNA and transcriptomic profiles in buffy coats of healthy subjects. We applied a linear model to relate a cumulative Z-Score of 6 PCB congeners, HCB and DDE (a breakdown product of DDT) to the miRNA profile which was subsequently integrated with the transcriptomic profile. Our study revealed a total of 93 miRNAs (53 positively and 40 negatively) to be significantly correlated with the exposure intensity (Z-Score) (Table 1). The top 4 positively correlated miRNAs, ranked by FDR (miR-193a-3p, miR-152, miR-31-5p and miR-34a-5p), have been described as tumor suppressor miRNAs^16–19^. We applied the same linear model to the individual POPs to see if the Z-Score of POPs represents the effects of all individual compounds. The analyses revealed that between 70 and 100 percent of the individual associations were also represented by the cumulative Z-Score of POPs (Supplementary Table S2).

Upon integration of the miRNA response with the transcriptomic profile we identified 217 significantly inverse regulated miRNA-gene pairs. The conducted gene-overrepresentation analysis of the inversely regulated genes revealed that mainly cancer-associated pathways, such as small cell lung cancer or chronic myeloid leukemia, or signaling pathways, like the wnt signaling pathway, were affected. Interestingly, also the thyroid hormone signaling and thyroid cancer pathways were affected (Table 2). The thyroid hormones are among the main suspects for human effects of POP exposure^20^.

Though unexpected, we have also observed 253 significantly positively correlated miRNA-gene pairs. The gene-overrepresentation analysis of the positively correlated genes revealed that mainly cancer-associated pathways, including chronic myeloid leukemia, or signaling pathways such as the p53 signaling pathway, were associated (Table 3). This finding is in contradiction to the generally expected repressive effect of miRNAs on target expression. Nevertheless, positive correlations have also been described in earlier studies on miRNA gene interactions, and thus the function of miRNAs may not be only repressive^21^. Since miRNAs are interwoven into complex regulatory networks, a suppression of a signaling mediator can lead to the transcription of a target gene, and consequently, result in a positive correlation^22^. Further studies are needed to unravel these complex interactions to provide a better understanding of the signaling networks involved. As these intermediates are unknown or have not been measured in this study, we focused mainly on the inverse correlations.

Among the downregulated miRNAs, miR-484 showed the most inverse regulations with genes derived from the KEGG pathways. The downregulation of miR-484 appeared to activate the expression and translation of the target oncogene *ABL1* and the Heat Shock Proteins *HSP90AA1* and *HSP90AB1*. The Abelson tyrosine kinases of the family *ABL* have been identified as key drivers of leukemia in humans. Activation of the proto-oncogene ABL1, a gene involved in signaling pathways that control cell growth and survival, and heat shock proteins of the *HSP90* family have been reported in many common cancer types^23, 24^.

We also found an increased expression in *TBL1XR1*, a gene playing an important role in the development of B-cell non-Hodgkin lymphomas^25^, in concordance with the downregulation of miR-501-3p and miR-502-3p. Furthermore, both miRNAs miR-320 and miR-486 have been reported to be downregulated in many types of cancer^26, 27^. In this study we observed miR-320 and 486-5p to be downregulated with the exposure and both activating the expression and translation of the forkhead box transcription Factor *FOXP1*. Increased abundance of *FOXP1* is known to enhance wnt signaling and is a predictor of poor prognosis and resistance to therapy in diffuse large B cell lymphoma^28^.

These interactions of miRNAs and oncogenes seem to promote the expression and possibly translation of genes involved in the hallmarks of cancer. However, next to these cancer risk increasing interactions our results have also shown miRNA-mediated repression of cancer related genes and therefore protection from carcinogenesis.

The upregulated miRNA miR-29a, a well-known tumor suppressor miRNA, had the highest number of significantly inverse correlations among the cancer-related gene targets^29^. In the present study this miRNA appears to repress a total of 8 gene targets, including the lymphoma-related genes *CCND1*, *BCL2*, *IGF1* and *VEGFA*. The protein encoded by *CCND1* (also known as *BCL1*) is a regulator of the cell cycle progression and plays an import role in cancer development. A recent study has shown that knocking down *CCND1* resulted in cell cycle arrest and induction of apoptosis^30^. Upregulation of the anti-apoptotic regulator *BCL2* was found in non-Hodgkin lymphoma and small cell lung cancer^31, 32^. Inactivation of *BCL2* is known to induce apoptosis and protects from cancer progression^33^. The Insulin-like growth factor *IGF1* is involved in cell proliferation, differentiation and apoptosis. High levels of *IGF1* have been found in several common cancers^34^. The vascular endothelial growth factor *VEGFA* induces angiogenesis by stimulating cell survival and proliferation. In cancer cells this gene promotes the formation of aggressive tumors^35^.

Furthermore, we observed an inverse correlation between the upregulated tumor suppressor miR-31-5p and its suppressed target, the proto-oncogene *SRC*. Elevations of the protein encoded by SRC have been described to induce cellular transformation, tumorigenicity, tumor progression, and metastasis^36^.

Altered expressions of miR-21 have been found in all common types of cancer and it has therefore been classified as an oncomir. Previous studies have shown that miR-21 plays a major role in the genesis of lymphoma^37^. In this study we identified both, miR-21-5p and miR-21-3p, to be upregulated with the intensity of exposure. The miRNA miR-21-5p showed a repressive effect on the anti-apoptotic *BCL2*, but also on the proto-oncogenes *TIAM1* (T-cell lymphoma invasion and metastasis 1) and *MYC*. Previous studies have reported that *TIAM1* modulates a number of cellular processes associated with tumor progression and overexpression of this gene has been found in various tumor types^38^.

The proto-oncogene *MYC* is strongly associated with lymphomas and adverse clinical outcomes related to B-cell malignancies. It is known to be the most commonly overexpressed oncogene in cancer and a robust prognostic marker for B-cell lymphomas. Recent evidence showed that there is significant crosstalk between *MYC* and miRNAs, with *MYC* also controlling the expression of a group of miRNAs. Repression of miRNAs by *MYC*, including tumor suppressor miRNAs like miR-29 and miR-34a, has been shown to contribute to cellular survival by activating anti-apoptotic proteins such as *CCND1* and *BCL2*. But also *MYC*-induced activation of miRNAs has been shown to promote cell cycle progression^39^. Our data showed a significant inverse correlation for the increased miR-21-5p and decreased *MYC*. However, In addition to the repressive effect of miR-21-5p on *MYC*, we observed five decreased miRNAs (let-7b-5p, miR-92a, miR-320b, miR-324-3p and miR-423-5p) to exhibit significant positive correlations with *MYC*. The roles of these miRNAs in carcinogenesis have not yet been fully understood. Nevertheless, these five decreased miRNAs could be suggested to have a potential stimulating role as regulators in the *MYC*-driven lymphomagenesis.

A recently published study demonstrated a signature of 128 miRNAs as potential novel diagnostic markers for B-cell lymphomas^40^. The herein presented exposure signature of 93 miRNAs showed an overlap of 28 (30%) miRNAs with the diagnostic lymphoma signature (including the tumor suppressor miRNAs mir-193, miR-152 and miR-34a). This overlap of miRNAs suggests similar pathways to be affected by lymphoma and the POP exposure. To assess whether the future lymphoma cases had an enrichment of risk-increasing miRNAs over the healthy controls, we performed a stratified analysis on only the future lymphoma cases and the healthy controls separately (Supplementary Table S3). The 91 associations of the future cases showed 31 (34%) miRNAs and the 128 associations of the healthy controls showed 54 (42%) miRNAs overlapping with the lymphoma signature. According to this overlap we did not see an overrepresentation of risk-increasing associations among the future lymphoma cases. A similar pattern was observed for the non-smokers showing 162 and the smoker with only 60 associations. The lower number of associations among the future cases and smokers might be a result of a higher biological variability with respect to miRNA expression among the future cases. Possibly, an early cancer and smoking increases the miRNA variability.

To our knowledge we have demonstrated the first evidence of alternations in the miRNA machinery upon environmental exposure to POPs in a population-based study. Unlike conventional approaches in cancer risk assessment we have shown that these miRNAs respond to a mixture of environmental carcinogens. Furthermore, we have shown that the interplay of the affected miRNA profile with the transcriptome involves genes essential for carcinogenesis. The miRNA and transcriptomic response to environmental carcinogens revealed that mechanisms are activated towards processes that possibly increase the risk of carcinogenesis, but also may be protective. Therefore, the observed miRNAs can be seen as key regulators of health and disease and have great potential to contribute to biomarker-based environmental health risk assessment.

## Methods

### Selection of the population

The Northern Sweden Health and Disease Study (NSHDS) comprises of 94,630 sampling occasions from 74,690 unique individuals. Within the EnviroGenomarkers project archived blood samples and exposure/health data were derived from -at that time- healthy subjects, including 229 future cases of B-cell lymphoma and 327 controls of the prospective NSHDS. No subject was diagnosed with lymphoma within less than two years of blood sample collection. Cases were matched to healthy controls by gender, age (+/− 2.5years), hospital and date of blood collection (+/− 6 months)^11^. For this study we randomly selected 226 buffy coat samples for integrated miRNA and transcriptome analysis (Table 4, Supplementary Figure S1). To determine the statistical power of the sample size we conducted a power calculation for microarray experiments using the R package “ssize.fdr”^41^. Accordingly, a minimum of 190 subjects were required to achieve 80% power. For the 207 subjects that were analyzed in our study we calculated 84% power at the 5% significance level (FDR corrected).The EnviroGenomarkers project and its associated studies and protocols were approved by the Regional Ethical Review Board of the Umea Division of Medical Research and all participants gave written informed consent. This study was conducted in accordance with the approved guidelines and regulations.

**Table 4:** Study population data.

Population			Age, mean (SD)	BMI, mean (SD)	Smoking status			Future lymphoma	
Total	Female	Male			Current	Former	Never	Case	Control
226	94	132	51.1 (7.6)	25.6 (5.3)	48	42	129	113	113

### Internal exposure assessment

Serum concentrations of 6 PCB congeners (PCB118, PCB153, PCB138, PCB156, PCB170 and PCB180), DDE and HCB were determined by means of a Agilent 6890 gas chromatographer connected to a Waters Autospec Ultima high resolution mass spectrometer as described in an earlier study on the exposure data^12, 13^. Per subject we calculated a *Z-Score*_*(POPs)*_ as a representative of the internal exposure to the mixture of POPs. The *Z-Score*_*(POPs*)_ was defined as the sum of the Z-Scores for each compound: *Z-Score*_*(compound)*_ = *(X − μ)/σ* (where *X* represents the value of the subject, *μ* the mean and *σ* the standard deviation of the population)^42^. Therefore, *Z-Score*_*(POPs)*_ = *Z-Score*_*(PCB118)*_ + *Z-Score*_*(PCB153)*_ + *Z-Score*_*(PCB138)*_ + *Z-Score*_*(PCB156)*_ + *Z-Score*_*(PCB170)*_ + *Z-Score*_*(PCB180)*_ + *Z-Score*_*(HCB)*_ + *Z-Score*_*(DDE)*_.

### Analytical procedures

Total RNA extraction from buffy coats, analysis of miRNA (Agilent 8 × 60K human miRNA microarray) and transcriptome profiling (Agilent 4 × 44K human whole genome microarray), and the corresponding data quality assessment and preprocessing were performed as described in an earlier publication^14^. These analyses provided expression data for 547 miRNAs and 15,805 genes. Due to insufficient quality and missing values in exposure/health data 19 subjects were excluded leaving 207 subjects for the data analysis.

### Statistical analysis

The miRNA and transcriptomic data was analyzed using the open-source software R (version 3.1.1) and Bioconductor^43^. The miRNA and transcriptomic raw signals were corrected for hybridization batch-effect as well as white blood cell counts (CD4, CD8, NK, B cells, monocytes, granulocytes) using ComBat (sva package version 3.18.0)^44^. The cell counts were derived from methylation data as described earlier^14^. We used a linear model provided by the R package limma (version 3.26.9) to determine miRNAs significantly associated with the exposure intensity (cumulative Z-Score) as described in the limma manual^45^. Within the analysis we additionally adjusted for the confounding variables sex, age, smoking status and future disease (future case or control). MicroRNAs were considered to be significantly associated with the Z-Score at a false discovery rate (FDR) below five percent^46^.

### Data integration and pathway analysis

From the experimentally validated microRNA-target interaction database miRTarBase (release 6) all gene-targets per exposure associated miRNA were retrieved^47^. A matrix for each miRNA consisting of the microarray signals of that particular miRNA and the signals of all its gene-targets was generated. Upon calculating the Pearson correlation using R package stats (version 3.2.2), all significantly correlating targets were selected for subsequent pathway analysis (r < −0.4 or > 0.4, FDR < 0.05). Pathway and Gene Ontology analyses of the target genes were performed by over-representation analysis in ConsensuthPathDB (Release 31)^48^ and gene set analysis using the Molecular Signatures Database (version 6.0)^15^. All genes linked to the KEGG pathways related to cancer were exported to Cytoscape (version 3.4.0) and visualized with their miRNA interactions.

## Electronic supplementary material

Supplementary Information
