# Supplementary material for: MicroRNA profile for health risk assessment: Environmental exposure to persistent organic pollutants strongly affects the human blood microRNA machinery
Source: Sci Rep. 2017 Aug 23;7:9262. doi: 10.1038/s41598-017-10167-7 (PMC5569060; doi:10.1038/s41598-017-10167-7)
Supplement: Supplementary file 1 — Supplementary Information [file 41598_2017_10167_MOESM1_ESM.doc]

# MicroRNA profile for health risk assessment: Environmental exposure to persistent organic pollutants strongly affects the human blood microRNA machinery

Julian Krauskopf^1*^, Theo M. de Kok^1^, Dennie G. Hebels^1^, Ingvar A. Bergdahl^2^, Anders Johansson^3^, Florentin Spaeth^4^, Hannu Kiviranta^5^, Panu Rantakokko^5^, Soterios A. Kyrtopoulos^6^, Jos C. Kleinjans^1^

**Supplementary Figures**


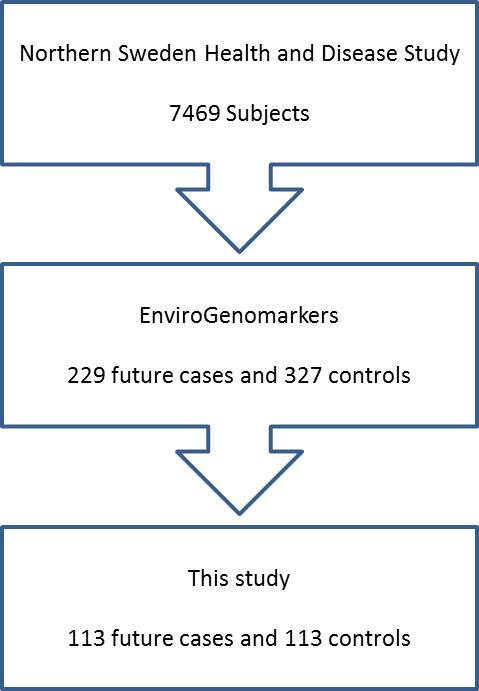


Figure S1: Study flow chart. The flow chart describes the selection of the subjects from the Northern Sweden Health and Disease Study, the EnviroGenomarkers project and the present study.

**Supplementary Tables**

Supplementary Table S1: List of miRNAs associated with the exposure intensity (sorted by ascending FDR)

| **miRNA** | **Regulation** | **FDR** |
| --- | --- | --- |
| hsa-miR-193a-3p | Up | 2.92E-03 |
| hsa-miR-152 | Up | 4.65E-03 |
| hsa-miR-31-5p | Up | 4.65E-03 |
| hsa-miR-532-3p | Down | 9.41E-03 |
| hsa-miR-324-3p | Down | 1.02E-02 |
| hsa-miR-320d | Down | 1.02E-02 |
| hsa-miR-320e | Down | 1.02E-02 |
| hsa-miR-486-5p | Down | 1.02E-02 |
| hsa-miR-34a-5p | Up | 1.02E-02 |
| hsa-miR-331-3p | Down | 1.02E-02 |
| hsa-miR-21-5p | Up | 1.02E-02 |
| hsa-miR-501-3p | Down | 1.02E-02 |
| hsa-miR-320c | Down | 1.02E-02 |
| hsa-miR-21-3p | Up | 1.02E-02 |
| hsa-miR-550a-3-5p | Down | 1.02E-02 |
| hsa-miR-29b-3p | Up | 1.02E-02 |
| hsa-miR-135a-3p | Up | 1.02E-02 |
| hsa-miR-29c-3p | Up | 1.07E-02 |
| hsa-miR-628-5p | Up | 1.07E-02 |
| hsa-miR-146b-5p | Up | 1.07E-02 |
| hsa-miR-423-5p | Down | 1.07E-02 |
| hsa-miR-4291 | Up | 1.07E-02 |
| hsa-miR-652-3p | Down | 1.07E-02 |
| hsa-miR-197-3p | Down | 1.07E-02 |
| hsa-miR-551b-3p | Up | 1.07E-02 |
| hsa-miR-25-5p | Down | 1.07E-02 |
| hsa-miR-92a-3p | Down | 1.07E-02 |
| hsa-miR-141-3p | Up | 1.07E-02 |
| hsa-miR-582-5p | Up | 1.15E-02 |
| hsa-miR-142-5p | Up | 1.15E-02 |
| hsa-miR-106b-5p | Up | 1.25E-02 |
| hsa-let-7c | Down | 1.28E-02 |
| hsa-miR-222-3p | Up | 1.28E-02 |
| hsa-miR-550a-3p | Down | 1.28E-02 |
| hsa-miR-361-3p | Down | 1.30E-02 |
| hsa-miR-320b | Down | 1.40E-02 |
| hsa-miR-501-5p | Down | 1.47E-02 |
| hsa-miR-4689 | Up | 1.47E-02 |
| hsa-miR-4323 | Down | 1.58E-02 |
| hsa-miR-340-5p | Up | 1.58E-02 |
| hsa-miR-301a-3p | Up | 1.58E-02 |
| hsa-miR-362-3p | Up | 1.58E-02 |
| hsa-miR-491-5p | Down | 1.58E-02 |
| hsa-miR-301b | Up | 1.88E-02 |
| hsa-let-7b-5p | Down | 1.88E-02 |
| hsa-miR-484 | Down | 1.98E-02 |
| hsa-miR-502-3p | Down | 1.98E-02 |
| hsa-miR-30d-5p | Down | 1.98E-02 |
| hsa-miR-505-5p | Down | 1.98E-02 |
| hsa-miR-4507 | Down | 1.98E-02 |
| hsa-miR-142-3p | Up | 1.98E-02 |
| hsa-miR-574-3p | Down | 1.98E-02 |
| hsa-miR-144-3p | Up | 2.02E-02 |
| hsa-miR-32-5p | Up | 2.02E-02 |
| hsa-miR-143-3p | Up | 2.03E-02 |
| hsa-miR-545-3p | Up | 2.03E-02 |
| hsa-miR-148a-3p | Up | 2.03E-02 |
| hsa-miR-744-5p | Down | 2.08E-02 |
| hsa-miR-425-5p | Down | 2.08E-02 |
| hsa-miR-1587 | Down | 2.17E-02 |
| hsa-miR-500a-3p | Down | 2.37E-02 |
| hsa-miR-338-3p | Up | 2.37E-02 |
| hsa-miR-101-3p | Up | 2.37E-02 |
| hsa-miR-548am-5p | Up | 2.37E-02 |
| hsa-miR-30e-5p | Up | 2.37E-02 |
| hsa-miR-126-5p | Up | 2.48E-02 |
| hsa-miR-766-3p | Down | 2.59E-02 |
| hsa-miR-4317 | Up | 2.65E-02 |
| hsa-miR-200c-3p | Down | 2.71E-02 |
| hsa-miR-7-1-3p | Up | 2.75E-02 |
| hsa-miR-598 | Up | 2.75E-02 |
| hsa-miR-27b-3p | Up | 2.75E-02 |
| hsa-miR-1307-5p | Up | 2.77E-02 |
| hsa-miR-199a-3p | Up | 2.84E-02 |
| hsa-miR-320a | Down | 2.85E-02 |
| hsa-miR-199a-5p | Up | 2.85E-02 |
| hsa-miR-140-5p | Up | 3.09E-02 |
| hsa-miR-103a-3p | Down | 3.09E-02 |
| hsa-miR-219-5p | Up | 3.09E-02 |
| hsa-miR-1270 | Down | 3.20E-02 |
| hsa-miR-1537 | Up | 3.21E-02 |
| hsa-miR-4732-3p | Down | 3.29E-02 |
| hsa-miR-33a-5p | Up | 3.29E-02 |
| hsa-miR-101-5p | Up | 3.44E-02 |
| hsa-miR-29a-3p | Up | 3.44E-02 |
| hsa-miR-424-5p | Up | 3.44E-02 |
| hsa-miR-30b-5p | Up | 3.80E-02 |
| hsa-miR-4505 | Down | 3.81E-02 |
| hsa-miR-335-5p | Up | 3.83E-02 |
| hsa-miR-296-5p | Down | 3.89E-02 |
| hsa-miR-376a-3p | Up | 3.98E-02 |
| hsa-miR-221-5p | Up | 3.98E-02 |
| hsa-miR-590-5p | Up | 3.98E-02 |

Supplementary Table S2: List of miRNAs associated with the individual POPs.

| PCB118 (pvalue<0.05) | PCB138 | PCB153 | PCB156 | PCB170 | PCB180 | HCB | DDE (pvalue<0.05) |
| --- | --- | --- | --- | --- | --- | --- | --- |
| hsa-let-7b-5p | hsa-let-7b-5p | hsa-let-7b-5p | hsa-miR-193a-3p | hsa-let-7c | hsa-let-7c | hsa-let-7b-5p | hsa-let-7b-5p |
| hsa-let-7c | hsa-let-7c | hsa-let-7c | hsa-miR-31-5p | hsa-miR-106b-5p | hsa-miR-10a-5p | hsa-let-7c | hsa-let-7c |
| hsa-let-7d-3p | hsa-miR-101-3p | hsa-miR-1 | hsa-miR-152 | hsa-miR-10a-5p | hsa-miR-135a-3p | hsa-let-7d-3p | hsa-miR-101-3p |
| hsa-miR-101-3p | hsa-miR-101-5p | hsa-miR-101-3p | hsa-miR-532-3p | hsa-miR-126-5p | hsa-miR-140-5p | hsa-miR-103a-3p | hsa-miR-101-5p |
| hsa-miR-101-5p | hsa-miR-106b-5p | hsa-miR-101-5p | hsa-miR-25-5p | hsa-miR-135a-3p | hsa-miR-141-3p | hsa-miR-106b-5p | hsa-miR-103a-3p |
| hsa-miR-103a-3p | hsa-miR-126-5p | hsa-miR-103a-3p | hsa-miR-34a-5p | hsa-miR-140-5p | hsa-miR-142-3p | hsa-miR-1238-3p | hsa-miR-106b-5p |
| hsa-miR-106b-5p | hsa-miR-1307-5p | hsa-miR-106b-5p | hsa-miR-628-5p | hsa-miR-141-3p | hsa-miR-142-5p | hsa-miR-1270 | hsa-miR-1181 |
| hsa-miR-126-5p | hsa-miR-135a-3p | hsa-miR-10a-5p | hsa-miR-486-5p | hsa-miR-142-3p | hsa-miR-143-3p | hsa-miR-132-3p | hsa-miR-1234-3p |
| hsa-miR-1304-3p | hsa-miR-140-5p | hsa-miR-126-5p | hsa-miR-21-3p | hsa-miR-142-5p | hsa-miR-146a-5p | hsa-miR-135a-3p | hsa-miR-1238-3p |
| hsa-miR-1307-5p | hsa-miR-141-3p | hsa-miR-1270 | hsa-miR-29b-3p | hsa-miR-143-3p | hsa-miR-146b-5p | hsa-miR-141-3p | hsa-miR-125a-5p |
| hsa-miR-135a-3p | hsa-miR-142-3p | hsa-miR-1307-5p | hsa-miR-320d | hsa-miR-146a-5p | hsa-miR-148a-3p | hsa-miR-146b-5p | hsa-miR-126-5p |
| hsa-miR-141-3p | hsa-miR-142-5p | hsa-miR-135a-3p | hsa-miR-135a-3p | hsa-miR-146b-5p | hsa-miR-152 | hsa-miR-152 | hsa-miR-1270 |
| hsa-miR-142-3p | hsa-miR-143-3p | hsa-miR-136-5p | hsa-miR-146b-5p | hsa-miR-148a-3p | hsa-miR-1587 | hsa-miR-15b-5p | hsa-miR-1304-3p |
| hsa-miR-142-5p | hsa-miR-144-3p | hsa-miR-140-5p | hsa-miR-491-5p | hsa-miR-152 | hsa-miR-181a-3p | hsa-miR-193a-3p | hsa-miR-1307-5p |
| hsa-miR-143-3p | hsa-miR-146b-5p | hsa-miR-141-3p | hsa-miR-141-3p | hsa-miR-1587 | hsa-miR-193a-3p | hsa-miR-197-3p | hsa-miR-132-3p |
| hsa-miR-144-3p | hsa-miR-148a-3p | hsa-miR-142-3p | hsa-miR-320e | hsa-miR-181a-3p | hsa-miR-199a-3p | hsa-miR-200c-3p | hsa-miR-135a-3p |
| hsa-miR-144-5p | hsa-miR-152 | hsa-miR-142-5p | hsa-miR-331-3p | hsa-miR-193a-3p | hsa-miR-199a-5p | hsa-miR-21-3p | hsa-miR-141-3p |
| hsa-miR-146b-5p | hsa-miR-1537 | hsa-miR-143-3p | hsa-miR-296-5p | hsa-miR-199a-3p | hsa-miR-21-3p | hsa-miR-21-5p | hsa-miR-142-3p |
| hsa-miR-148a-3p | hsa-miR-1587 | hsa-miR-144-3p | hsa-miR-222-3p | hsa-miR-199a-5p | hsa-miR-21-5p | hsa-miR-222-3p | hsa-miR-142-5p |
| hsa-miR-152 | hsa-miR-193a-3p | hsa-miR-146a-5p | hsa-miR-501-3p | hsa-miR-19b-1-5p | hsa-miR-221-3p | hsa-miR-25-5p | hsa-miR-143-3p |
| hsa-miR-1537 | hsa-miR-197-3p | hsa-miR-146b-5p | hsa-miR-21-5p | hsa-miR-21-3p | hsa-miR-221-5p | hsa-miR-26a-5p | hsa-miR-144-3p |
| hsa-miR-15a-3p | hsa-miR-199a-3p | hsa-miR-148a-3p | hsa-miR-423-5p | hsa-miR-21-5p | hsa-miR-222-3p | hsa-miR-29b-3p | hsa-miR-152 |
| hsa-miR-15a-5p | hsa-miR-199a-5p | hsa-miR-152 | hsa-miR-4291 | hsa-miR-221-3p | hsa-miR-24-3p | hsa-miR-29c-3p | hsa-miR-1587 |
| hsa-miR-181a-5p | hsa-miR-199b-5p | hsa-miR-1537 | hsa-miR-92a-3p | hsa-miR-221-5p | hsa-miR-25-5p | hsa-miR-30d-5p | hsa-miR-15b-5p |
| hsa-miR-190a | hsa-miR-19a-3p | hsa-miR-1587 | hsa-miR-4689 | hsa-miR-222-3p | hsa-miR-27a-3p | hsa-miR-31-5p | hsa-miR-181a-5p |
| hsa-miR-193a-3p | hsa-miR-19b-3p | hsa-miR-15a-3p | hsa-miR-142-5p | hsa-miR-25-5p | hsa-miR-27b-3p | hsa-miR-320b | hsa-miR-193a-3p |
| hsa-miR-197-3p | hsa-miR-21-3p | hsa-miR-181a-3p | hsa-miR-29c-3p | hsa-miR-27a-3p | hsa-miR-296-5p | hsa-miR-320c | hsa-miR-197-3p |
| hsa-miR-199a-3p | hsa-miR-21-5p | hsa-miR-181d | hsa-miR-320c | hsa-miR-27b-3p | hsa-miR-29a-3p | hsa-miR-320d | hsa-miR-200c-3p |
| hsa-miR-199b-5p | hsa-miR-219-5p | hsa-miR-190a | hsa-miR-582-5p | hsa-miR-296-5p | hsa-miR-29b-3p | hsa-miR-320e | hsa-miR-21-5p |
| hsa-miR-19a-3p | hsa-miR-221-5p | hsa-miR-193a-3p | hsa-miR-551b-3p | hsa-miR-29a-3p | hsa-miR-29c-3p | hsa-miR-324-3p | hsa-miR-219-5p |
| hsa-miR-19b-3p | hsa-miR-222-3p | hsa-miR-197-3p | hsa-miR-324-3p | hsa-miR-29b-3p | hsa-miR-301a-3p | hsa-miR-328 | hsa-miR-222-3p |
| hsa-miR-200c-3p | hsa-miR-25-5p | hsa-miR-199a-3p | hsa-miR-652-3p | hsa-miR-29c-3p | hsa-miR-301b | hsa-miR-331-3p | hsa-miR-299-5p |
| hsa-miR-21-3p | hsa-miR-27b-3p | hsa-miR-199a-5p |  | hsa-miR-301a-3p | hsa-miR-30d-5p | hsa-miR-340-5p | hsa-miR-29b-3p |
| hsa-miR-21-5p | hsa-miR-29b-3p | hsa-miR-199b-5p |  | hsa-miR-301b | hsa-miR-31-5p | hsa-miR-34a-5p | hsa-miR-29c-3p |
| hsa-miR-222-3p | hsa-miR-29c-3p | hsa-miR-19a-3p |  | hsa-miR-30d-5p | hsa-miR-320b | hsa-miR-361-3p | hsa-miR-301a-3p |
| hsa-miR-223-3p | hsa-miR-301a-3p | hsa-miR-19b-1-5p |  | hsa-miR-30e-5p | hsa-miR-320c | hsa-miR-423-5p | hsa-miR-301b |
| hsa-miR-24-3p | hsa-miR-301b | hsa-miR-19b-3p |  | hsa-miR-31-5p | hsa-miR-320d | hsa-miR-425-5p | hsa-miR-30d-5p |
| hsa-miR-25-5p | hsa-miR-30b-5p | hsa-miR-200b-3p |  | hsa-miR-320a | hsa-miR-320e | hsa-miR-4257 | hsa-miR-30e-5p |
| hsa-miR-26a-5p | hsa-miR-30d-5p | hsa-miR-21-3p |  | hsa-miR-320b | hsa-miR-324-3p | hsa-miR-4291 | hsa-miR-3138 |
| hsa-miR-26b-5p | hsa-miR-30e-5p | hsa-miR-21-5p |  | hsa-miR-320c | hsa-miR-331-3p | hsa-miR-4318 | hsa-miR-31-5p |
| hsa-miR-27a-3p | hsa-miR-31-5p | hsa-miR-219-5p |  | hsa-miR-320d | hsa-miR-338-3p | hsa-miR-4323 | hsa-miR-3188 |
| hsa-miR-27b-3p | hsa-miR-320a | hsa-miR-221-3p |  | hsa-miR-320e | hsa-miR-33a-5p | hsa-miR-4513 | hsa-miR-320a |
| hsa-miR-29b-3p | hsa-miR-320b | hsa-miR-221-5p |  | hsa-miR-324-3p | hsa-miR-340-5p | hsa-miR-4651 | hsa-miR-320b |
| hsa-miR-29c-3p | hsa-miR-320c | hsa-miR-222-3p |  | hsa-miR-32-5p | hsa-miR-34a-5p | hsa-miR-4667-5p | hsa-miR-320c |
| hsa-miR-301a-3p | hsa-miR-320d | hsa-miR-223-3p |  | hsa-miR-331-3p | hsa-miR-361-3p | hsa-miR-4689 | hsa-miR-320d |
| hsa-miR-301b | hsa-miR-320e | hsa-miR-24-3p |  | hsa-miR-338-3p | hsa-miR-362-3p | hsa-miR-4697-5p | hsa-miR-320e |
| hsa-miR-30b-5p | hsa-miR-324-3p | hsa-miR-25-5p |  | hsa-miR-33a-5p | hsa-miR-3656 | hsa-miR-4732-3p | hsa-miR-324-3p |
| hsa-miR-30d-5p | hsa-miR-32-5p | hsa-miR-26a-5p |  | hsa-miR-340-5p | hsa-miR-3663-3p | hsa-miR-484 | hsa-miR-32-5p |
| hsa-miR-30e-5p | hsa-miR-331-3p | hsa-miR-27a-3p |  | hsa-miR-34a-5p | hsa-miR-376a-3p | hsa-miR-486-5p | hsa-miR-331-3p |
| hsa-miR-31-5p | hsa-miR-335-5p | hsa-miR-27b-3p |  | hsa-miR-361-3p | hsa-miR-376c-3p | hsa-miR-491-5p | hsa-miR-34a-5p |
| hsa-miR-3162-3p | hsa-miR-338-3p | hsa-miR-296-5p |  | hsa-miR-362-3p | hsa-miR-377-3p | hsa-miR-500a-3p | hsa-miR-361-3p |
| hsa-miR-3180-3p | hsa-miR-33a-5p | hsa-miR-29a-3p |  | hsa-miR-3656 | hsa-miR-423-5p | hsa-miR-501-3p | hsa-miR-362-3p |
| hsa-miR-320a | hsa-miR-340-5p | hsa-miR-29b-3p |  | hsa-miR-3663-3p | hsa-miR-4291 | hsa-miR-501-5p | hsa-miR-409-5p |
| hsa-miR-320b | hsa-miR-34a-5p | hsa-miR-29c-3p |  | hsa-miR-376a-3p | hsa-miR-4317 | hsa-miR-502-3p | hsa-miR-423-5p |
| hsa-miR-320c | hsa-miR-362-3p | hsa-miR-301a-3p |  | hsa-miR-377-3p | hsa-miR-4323 | hsa-miR-505-5p | hsa-miR-424-5p |
| hsa-miR-320d | hsa-miR-423-5p | hsa-miR-301b |  | hsa-miR-423-5p | hsa-miR-4433-3p | hsa-miR-532-3p | hsa-miR-425-5p |
| hsa-miR-320e | hsa-miR-424-5p | hsa-miR-30b-5p |  | hsa-miR-424-5p | hsa-miR-4505 | hsa-miR-550a-3-5p | hsa-miR-4323 |
| hsa-miR-324-3p | hsa-miR-425-5p | hsa-miR-30d-5p |  | hsa-miR-425-5p | hsa-miR-4507 | hsa-miR-550a-3p | hsa-miR-4505 |
| hsa-miR-32-5p | hsa-miR-4291 | hsa-miR-30e-5p |  | hsa-miR-4291 | hsa-miR-4689 | hsa-miR-550b-2-5p | hsa-miR-4507 |
| hsa-miR-328 | hsa-miR-4317 | hsa-miR-31-5p |  | hsa-miR-4317 | hsa-miR-4690-5p | hsa-miR-574-3p | hsa-miR-4513 |
| hsa-miR-331-3p | hsa-miR-4323 | hsa-miR-320a |  | hsa-miR-4323 | hsa-miR-4732-3p | hsa-miR-582-5p | hsa-miR-4651 |
| hsa-miR-335-5p | hsa-miR-4505 | hsa-miR-320b |  | hsa-miR-4433-3p | hsa-miR-484 | hsa-miR-625-5p | hsa-miR-4689 |
| hsa-miR-338-3p | hsa-miR-4507 | hsa-miR-320c |  | hsa-miR-4505 | hsa-miR-486-5p | hsa-miR-642a-3p | hsa-miR-4734 |
| hsa-miR-340-5p | hsa-miR-4689 | hsa-miR-320d |  | hsa-miR-4507 | hsa-miR-491-5p | hsa-miR-652-3p | hsa-miR-4737 |
| hsa-miR-34a-5p | hsa-miR-484 | hsa-miR-320e |  | hsa-miR-4689 | hsa-miR-500a-3p | hsa-miR-744-5p | hsa-miR-484 |
| hsa-miR-361-3p | hsa-miR-486-5p | hsa-miR-324-3p |  | hsa-miR-4690-5p | hsa-miR-501-3p | hsa-miR-766-3p | hsa-miR-486-5p |
| hsa-miR-362-3p | hsa-miR-491-5p | hsa-miR-32-5p |  | hsa-miR-4732-3p | hsa-miR-501-5p | hsa-miR-92a-3p | hsa-miR-491-5p |
| hsa-miR-374a-5p | hsa-miR-501-3p | hsa-miR-331-3p |  | hsa-miR-484 | hsa-miR-502-3p |  | hsa-miR-501-3p |
| hsa-miR-378g | hsa-miR-501-5p | hsa-miR-335-5p |  | hsa-miR-486-5p | hsa-miR-505-5p |  | hsa-miR-505-5p |
| hsa-miR-423-5p | hsa-miR-502-3p | hsa-miR-338-3p |  | hsa-miR-491-5p | hsa-miR-532-3p |  | hsa-miR-532-3p |
| hsa-miR-424-5p | hsa-miR-532-3p | hsa-miR-33a-5p |  | hsa-miR-500a-3p | hsa-miR-548am-5p |  | hsa-miR-550a-3-5p |
| hsa-miR-425-5p | hsa-miR-542-3p | hsa-miR-340-5p |  | hsa-miR-501-3p | hsa-miR-550a-3-5p |  | hsa-miR-550a-3p |
| hsa-miR-4257 | hsa-miR-545-3p | hsa-miR-34a-5p |  | hsa-miR-501-5p | hsa-miR-550a-3p |  | hsa-miR-551b-3p |
| hsa-miR-4291 | hsa-miR-550a-3-5p | hsa-miR-361-3p |  | hsa-miR-502-3p | hsa-miR-551b-3p |  | hsa-miR-574-3p |
| hsa-miR-4313 | hsa-miR-550a-3p | hsa-miR-362-3p |  | hsa-miR-505-5p | hsa-miR-574-3p |  | hsa-miR-579 |
| hsa-miR-4317 | hsa-miR-551b-3p | hsa-miR-3656 |  | hsa-miR-532-3p | hsa-miR-582-5p |  | hsa-miR-582-5p |
| hsa-miR-4323 | hsa-miR-582-5p | hsa-miR-376a-3p |  | hsa-miR-542-3p | hsa-miR-598 |  | hsa-miR-6134 |
| hsa-miR-4513 | hsa-miR-590-5p | hsa-miR-376c-3p |  | hsa-miR-545-3p | hsa-miR-628-5p |  | hsa-miR-625-5p |
| hsa-miR-451a | hsa-miR-598 | hsa-miR-377-3p |  | hsa-miR-548am-5p | hsa-miR-652-3p |  | hsa-miR-6508-5p |
| hsa-miR-4633-5p | hsa-miR-628-5p | hsa-miR-423-5p |  | hsa-miR-550a-3-5p | hsa-miR-6724-5p |  | hsa-miR-652-3p |
| hsa-miR-4651 | hsa-miR-652-3p | hsa-miR-424-5p |  | hsa-miR-550a-3p | hsa-miR-7-1-3p |  | hsa-miR-652-5p |
| hsa-miR-4656 | hsa-miR-7-1-3p | hsa-miR-425-5p |  | hsa-miR-551b-3p | hsa-miR-92a-3p |  | hsa-miR-744-5p |
| hsa-miR-4665-3p | hsa-miR-770-5p | hsa-miR-4291 |  | hsa-miR-574-3p |  |  | hsa-miR-766-3p |
| hsa-miR-4689 | hsa-miR-92a-3p | hsa-miR-4317 |  | hsa-miR-582-5p |  |  | hsa-miR-92a-3p |
| hsa-miR-484 |  | hsa-miR-4323 |  | hsa-miR-598 |  |  |  |
| hsa-miR-485-3p |  | hsa-miR-4433-3p |  | hsa-miR-628-5p |  |  |  |
| hsa-miR-486-5p |  | hsa-miR-4505 |  | hsa-miR-652-3p |  |  |  |
| hsa-miR-491-5p |  | hsa-miR-4507 |  | hsa-miR-7-1-3p |  |  |  |
| hsa-miR-497-5p |  | hsa-miR-450a-5p |  | hsa-miR-92a-3p |  |  |  |
| hsa-miR-500a-3p |  | hsa-miR-4689 |  | hsa-miR-93-5p |  |  |  |
| hsa-miR-501-3p |  | hsa-miR-4690-5p |  |  |  |  |  |
| hsa-miR-501-5p |  | hsa-miR-4732-3p |  |  |  |  |  |
| hsa-miR-502-3p |  | hsa-miR-4737 |  |  |  |  |  |
| hsa-miR-505-5p |  | hsa-miR-484 |  |  |  |  |  |
| hsa-miR-532-3p |  | hsa-miR-486-5p |  |  |  |  |  |
| hsa-miR-545-3p |  | hsa-miR-491-5p |  |  |  |  |  |
| hsa-miR-550a-3-5p |  | hsa-miR-500a-3p |  |  |  |  |  |
| hsa-miR-550a-3p |  | hsa-miR-501-3p |  |  |  |  |  |
| hsa-miR-551b-3p |  | hsa-miR-501-5p |  |  |  |  |  |
| hsa-miR-574-3p |  | hsa-miR-502-3p |  |  |  |  |  |
| hsa-miR-582-5p |  | hsa-miR-505-5p |  |  |  |  |  |
| hsa-miR-598 |  | hsa-miR-532-3p |  |  |  |  |  |
| hsa-miR-628-5p |  | hsa-miR-542-3p |  |  |  |  |  |
| hsa-miR-642a-3p |  | hsa-miR-545-3p |  |  |  |  |  |
| hsa-miR-6508-5p |  | hsa-miR-548am-5p |  |  |  |  |  |
| hsa-miR-652-3p |  | hsa-miR-550a-3-5p |  |  |  |  |  |
| hsa-miR-652-5p |  | hsa-miR-550a-3p |  |  |  |  |  |
| hsa-miR-7-1-3p |  | hsa-miR-551b-3p |  |  |  |  |  |
| hsa-miR-744-5p |  | hsa-miR-574-3p |  |  |  |  |  |
| hsa-miR-766-3p |  | hsa-miR-582-5p |  |  |  |  |  |
| hsa-miR-770-5p |  | hsa-miR-590-5p |  |  |  |  |  |
| hsa-miR-92a-3p |  | hsa-miR-598 |  |  |  |  |  |
|  |  | hsa-miR-628-5p |  |  |  |  |  |
|  |  | hsa-miR-652-3p |  |  |  |  |  |
|  |  | hsa-miR-652-5p |  |  |  |  |  |
|  |  | hsa-miR-6724-5p |  |  |  |  |  |
|  |  | hsa-miR-7-1-3p |  |  |  |  |  |
|  |  | hsa-miR-744-5p |  |  |  |  |  |
|  |  | hsa-miR-770-5p |  |  |  |  |  |
|  |  | hsa-miR-92a-3p |  |  |  |  |  |
|  |  | hsa-miR-93-5p |  |  |  |  |  |

Supplementary Table S3: List of miRNAs associated with the exposure intensity in stratified analysis.

| Full data(n = 207) | males(122) | | females(85) | | smokers (46) | | non-smokers(161) | | healthy controls (100) | | future cases (107) | |
| --- | --- | --- | --- | --- | --- | --- | --- | --- | --- | --- | --- | --- |
| FDR<0.05 | FDR<0.05 | pvalue<0.05 | FDR<0.05 | pvalue<0.05 | FDR<0.05 | pvalue<0.05 | FDR<0.05 | pvalue<0.05 | FDR<0.05 | pvalue<0.05 | FDR<0.05 | pvalue<0.05 |
| hsa-let-7b-5p | - | hsa-let-7b-5p | - | hsa-let-7c | - | hsa-let-7c | hsa-miR-106b-5p | hsa-let-7b-5p | hsa-let-7c | hsa-let-7b-5p | - | hsa-let-7g-5p |
| hsa-let-7c |  | hsa-let-7c |  | hsa-miR-1 |  | hsa-let-7d-5p | hsa-miR-1304-3p | hsa-let-7c | hsa-miR-1307-5p | hsa-let-7c |  | hsa-miR-100-5p |
| hsa-miR-101-3p |  | hsa-let-7d-3p |  | hsa-miR-101-5p |  | hsa-miR-1234-5p | hsa-miR-146b-5p | hsa-let-7d-3p | hsa-miR-141-3p | hsa-let-7d-3p |  | hsa-miR-10a-5p |
| hsa-miR-101-5p |  | hsa-miR-101-3p |  | hsa-miR-103a-3p |  | hsa-miR-1236-5p | hsa-miR-148a-3p | hsa-let-7g-5p | hsa-miR-142-5p | hsa-let-7d-5p |  | hsa-miR-126-3p |
| hsa-miR-103a-3p |  | hsa-miR-106b-5p |  | hsa-miR-1236-5p |  | hsa-miR-1255b-5p | hsa-miR-152 | hsa-miR-1 | hsa-miR-144-3p | hsa-miR-1 |  | hsa-miR-126-5p |
| hsa-miR-106b-5p |  | hsa-miR-1234-3p |  | hsa-miR-1255b-5p |  | hsa-miR-132-3p | hsa-miR-193a-3p | hsa-miR-101-3p | hsa-miR-193a-3p | hsa-miR-101-3p |  | hsa-miR-128 |
| hsa-miR-126-5p |  | hsa-miR-126-5p |  | hsa-miR-1270 |  | hsa-miR-142-5p | hsa-miR-197-3p | hsa-miR-101-5p | hsa-miR-197-3p | hsa-miR-101-5p |  | hsa-miR-1281 |
| hsa-miR-1270 |  | hsa-miR-1281 |  | hsa-miR-132-3p |  | hsa-miR-15b-5p | hsa-miR-21-5p | hsa-miR-103a-3p | hsa-miR-21-5p | hsa-miR-103a-3p |  | hsa-miR-133b |
| hsa-miR-1307-5p |  | hsa-miR-1304-3p |  | hsa-miR-135a-3p |  | hsa-miR-183-5p | hsa-miR-29c-3p | hsa-miR-106b-5p | hsa-miR-222-3p | hsa-miR-106b-5p |  | hsa-miR-135a-3p |
| hsa-miR-135a-3p |  | hsa-miR-1307-5p |  | hsa-miR-140-5p |  | hsa-miR-192-3p | hsa-miR-30b-5p | hsa-miR-10a-5p | hsa-miR-25-5p | hsa-miR-1181 |  | hsa-miR-140-5p |
| hsa-miR-140-5p |  | hsa-miR-135a-3p |  | hsa-miR-141-3p |  | hsa-miR-193a-3p | hsa-miR-31-5p | hsa-miR-1228-3p | hsa-miR-296-5p | hsa-miR-1202 |  | hsa-miR-146a-5p |
| hsa-miR-141-3p |  | hsa-miR-140-5p |  | hsa-miR-142-5p |  | hsa-miR-21-3p | hsa-miR-320d | hsa-miR-1234-3p | hsa-miR-29b-3p | hsa-miR-1225-5p |  | hsa-miR-146b-5p |
| hsa-miR-142-3p |  | hsa-miR-141-3p |  | hsa-miR-143-3p |  | hsa-miR-25-3p | hsa-miR-320e | hsa-miR-1238-3p | hsa-miR-29c-3p | hsa-miR-1255b-5p |  | hsa-miR-148a-3p |
| hsa-miR-142-5p |  | hsa-miR-142-3p |  | hsa-miR-146a-5p |  | hsa-miR-29a-3p | hsa-miR-324-3p | hsa-miR-126-3p | hsa-miR-31-5p | hsa-miR-1270 |  | hsa-miR-148b-3p |
| hsa-miR-143-3p |  | hsa-miR-142-5p |  | hsa-miR-146b-5p |  | hsa-miR-29b-1-5p | hsa-miR-340-5p | hsa-miR-126-5p | hsa-miR-320c | hsa-miR-1301 |  | hsa-miR-151a-3p |
| hsa-miR-144-3p |  | hsa-miR-144-3p |  | hsa-miR-152 |  | hsa-miR-29b-3p | hsa-miR-34a-5p | hsa-miR-1270 | hsa-miR-320d | hsa-miR-1307-5p |  | hsa-miR-152 |
| hsa-miR-146b-5p |  | hsa-miR-146b-5p |  | hsa-miR-155-5p |  | hsa-miR-31-5p | hsa-miR-423-5p | hsa-miR-1273f | hsa-miR-320e | hsa-miR-130b-3p |  | hsa-miR-1587 |
| hsa-miR-148a-3p |  | hsa-miR-148a-3p |  | hsa-miR-15a-3p |  | hsa-miR-3163 | hsa-miR-4323 | hsa-miR-1304-3p | hsa-miR-324-3p | hsa-miR-132-3p |  | hsa-miR-15a-3p |
| hsa-miR-152 |  | hsa-miR-152 |  | hsa-miR-182-3p |  | hsa-miR-3188 | hsa-miR-4507 | hsa-miR-1307-5p | hsa-miR-331-3p | hsa-miR-135a-3p |  | hsa-miR-191-5p |
| hsa-miR-1537 |  | hsa-miR-1537 |  | hsa-miR-193a-3p |  | hsa-miR-3200-5p | hsa-miR-4513 | hsa-miR-133b | hsa-miR-33a-5p | hsa-miR-140-3p |  | hsa-miR-192-5p |
| hsa-miR-1587 |  | hsa-miR-1587 |  | hsa-miR-199a-3p |  | hsa-miR-320c | hsa-miR-486-5p | hsa-miR-135a-3p | hsa-miR-423-5p | hsa-miR-141-3p |  | hsa-miR-193a-3p |
| hsa-miR-193a-3p |  | hsa-miR-15a-5p |  | hsa-miR-199a-5p |  | hsa-miR-320e | hsa-miR-532-3p | hsa-miR-136-5p | hsa-miR-425-5p | hsa-miR-142-3p |  | hsa-miR-196b-5p |
| hsa-miR-197-3p |  | hsa-miR-181a-3p |  | hsa-miR-199b-5p |  | hsa-miR-331-3p | hsa-miR-548am-5p | hsa-miR-140-5p | hsa-miR-4513 | hsa-miR-142-5p |  | hsa-miR-199a-3p |
| hsa-miR-199a-3p |  | hsa-miR-181a-5p |  | hsa-miR-21-3p |  | hsa-miR-34a-5p | hsa-miR-551b-3p | hsa-miR-141-3p | hsa-miR-484 | hsa-miR-143-3p |  | hsa-miR-199a-5p |
| hsa-miR-199a-5p |  | hsa-miR-181d |  | hsa-miR-21-5p |  | hsa-miR-3607-3p | hsa-miR-628-5p | hsa-miR-142-3p | hsa-miR-486-5p | hsa-miR-144-3p |  | hsa-miR-21-3p |
| hsa-miR-200c-3p |  | hsa-miR-1827 |  | hsa-miR-221-5p |  | hsa-miR-363-3p |  | hsa-miR-142-5p | hsa-miR-500a-3p | hsa-miR-146b-5p |  | hsa-miR-221-3p |
| hsa-miR-21-3p |  | hsa-miR-190a |  | hsa-miR-223-3p |  | hsa-miR-3653 |  | hsa-miR-143-3p | hsa-miR-501-3p | hsa-miR-152 |  | hsa-miR-221-5p |
| hsa-miR-21-5p |  | hsa-miR-191-3p |  | hsa-miR-22-3p |  | hsa-miR-3663-3p |  | hsa-miR-144-3p | hsa-miR-502-3p | hsa-miR-1537 |  | hsa-miR-223-3p |
| hsa-miR-219-5p |  | hsa-miR-193a-3p |  | hsa-miR-23a-3p |  | hsa-miR-3676-5p |  | hsa-miR-144-5p | hsa-miR-532-3p | hsa-miR-1587 |  | hsa-miR-224-5p |
| hsa-miR-221-5p |  | hsa-miR-197-3p |  | hsa-miR-24-3p |  | hsa-miR-425-5p |  | hsa-miR-146a-5p | hsa-miR-542-3p | hsa-miR-15b-5p |  | hsa-miR-23a-3p |
| hsa-miR-222-3p |  | hsa-miR-19a-3p |  | hsa-miR-25-3p |  | hsa-miR-4281 |  | hsa-miR-146b-5p | hsa-miR-550a-3-5p | hsa-miR-181a-5p |  | hsa-miR-23b-3p |
| hsa-miR-25-5p |  | hsa-miR-19b-3p |  | hsa-miR-25-5p |  | hsa-miR-4284 |  | hsa-miR-148a-3p | hsa-miR-550a-3p | hsa-miR-183-3p |  | hsa-miR-24-3p |
| hsa-miR-27b-3p |  | hsa-miR-200b-3p |  | hsa-miR-26a-5p |  | hsa-miR-4286 |  | hsa-miR-148b-3p | hsa-miR-574-3p | hsa-miR-183-5p |  | hsa-miR-25-5p |
| hsa-miR-296-5p |  | hsa-miR-200c-3p |  | hsa-miR-27a-3p |  | hsa-miR-4318 |  | hsa-miR-152 | hsa-miR-652-3p | hsa-miR-190a |  | hsa-miR-26b-5p |
| hsa-miR-29a-3p |  | hsa-miR-202-3p |  | hsa-miR-27b-3p |  | hsa-miR-4455 |  | hsa-miR-1537 | hsa-miR-92a-3p | hsa-miR-191-5p |  | hsa-miR-27a-3p |
| hsa-miR-29b-3p |  | hsa-miR-21-3p |  | hsa-miR-29a-3p |  | hsa-miR-4516 |  | hsa-miR-1587 | hsa-miR-942 | hsa-miR-193a-3p |  | hsa-miR-27b-3p |
| hsa-miR-29c-3p |  | hsa-miR-21-5p |  | hsa-miR-29b-1-5p |  | hsa-miR-4707-3p |  | hsa-miR-15a-3p |  | hsa-miR-197-3p |  | hsa-miR-29b-1-5p |
| hsa-miR-301a-3p |  | hsa-miR-219-5p |  | hsa-miR-29b-3p |  | hsa-miR-4732-3p |  | hsa-miR-15a-5p |  | hsa-miR-199b-5p |  | hsa-miR-301b |
| hsa-miR-301b |  | hsa-miR-221-5p |  | hsa-miR-29c-3p |  | hsa-miR-4732-5p |  | hsa-miR-17-3p |  | hsa-miR-19a-3p |  | hsa-miR-30b-5p |
| hsa-miR-30b-5p |  | hsa-miR-222-3p |  | hsa-miR-301a-3p |  | hsa-miR-4734 |  | hsa-miR-181a-3p |  | hsa-miR-19b-1-5p |  | hsa-miR-30d-5p |
| hsa-miR-30d-5p |  | hsa-miR-24-1-5p |  | hsa-miR-301b |  | hsa-miR-4739 |  | hsa-miR-181a-5p |  | hsa-miR-19b-3p |  | hsa-miR-30e-3p |
| hsa-miR-30e-5p |  | hsa-miR-25-5p |  | hsa-miR-30d-5p |  | hsa-miR-4745-5p |  | hsa-miR-181d |  | hsa-miR-200c-3p |  | hsa-miR-31-5p |
| hsa-miR-31-5p |  | hsa-miR-26b-5p |  | hsa-miR-3127-5p |  | hsa-miR-4746-3p |  | hsa-miR-18a-5p |  | hsa-miR-202-3p |  | hsa-miR-320b |
| hsa-miR-320a |  | hsa-miR-296-5p |  | hsa-miR-31-5p |  | hsa-miR-4763-3p |  | hsa-miR-190a |  | hsa-miR-21-3p |  | hsa-miR-320c |
| hsa-miR-320b |  | hsa-miR-29b-3p |  | hsa-miR-3163 |  | hsa-miR-486-3p |  | hsa-miR-191-3p |  | hsa-miR-21-5p |  | hsa-miR-320d |
| hsa-miR-320c |  | hsa-miR-29c-3p |  | hsa-miR-3176 |  | hsa-miR-5003-5p |  | hsa-miR-192-3p |  | hsa-miR-219-5p |  | hsa-miR-320e |
| hsa-miR-320d |  | hsa-miR-301a-3p |  | hsa-miR-320b |  | hsa-miR-5010-5p |  | hsa-miR-192-5p |  | hsa-miR-222-3p |  | hsa-miR-335-5p |
| hsa-miR-320e |  | hsa-miR-301b |  | hsa-miR-320c |  | hsa-miR-505-5p |  | hsa-miR-193a-3p |  | hsa-miR-24-1-5p |  | hsa-miR-338-3p |
| hsa-miR-324-3p |  | hsa-miR-30b-5p |  | hsa-miR-320d |  | hsa-miR-5189 |  | hsa-miR-196b-5p |  | hsa-miR-25-5p |  | hsa-miR-339-3p |
| hsa-miR-32-5p |  | hsa-miR-30d-5p |  | hsa-miR-320e |  | hsa-miR-532-3p |  | hsa-miR-197-3p |  | hsa-miR-26b-3p |  | hsa-miR-340-3p |
| hsa-miR-331-3p |  | hsa-miR-30e-5p |  | hsa-miR-324-3p |  | hsa-miR-550a-3-5p |  | hsa-miR-199a-3p |  | hsa-miR-296-5p |  | hsa-miR-340-5p |
| hsa-miR-335-5p |  | hsa-miR-3138 |  | hsa-miR-331-3p |  | hsa-miR-550a-3p |  | hsa-miR-199a-5p |  | hsa-miR-29a-3p |  | hsa-miR-34a-5p |
| hsa-miR-338-3p |  | hsa-miR-31-5p |  | hsa-miR-338-3p |  | hsa-miR-550b-2-5p |  | hsa-miR-199b-5p |  | hsa-miR-29b-3p |  | hsa-miR-361-3p |
| hsa-miR-33a-5p |  | hsa-miR-320a |  | hsa-miR-339-3p |  | hsa-miR-582-5p |  | hsa-miR-19a-3p |  | hsa-miR-29c-3p |  | hsa-miR-362-3p |
| hsa-miR-340-5p |  | hsa-miR-320b |  | hsa-miR-340-5p |  | hsa-miR-6090 |  | hsa-miR-19b-1-5p |  | hsa-miR-301a-3p |  | hsa-miR-374a-5p |
| hsa-miR-34a-5p |  | hsa-miR-320c |  | hsa-miR-342-3p |  | hsa-miR-652-3p |  | hsa-miR-19b-3p |  | hsa-miR-301b |  | hsa-miR-374b-5p |
| hsa-miR-361-3p |  | hsa-miR-320d |  | hsa-miR-34a-5p |  | hsa-miR-6723-5p |  | hsa-miR-200b-3p |  | hsa-miR-30d-5p |  | hsa-miR-374c-5p |
| hsa-miR-362-3p |  | hsa-miR-320e |  | hsa-miR-361-3p |  | hsa-miR-93-5p |  | hsa-miR-200c-3p |  | hsa-miR-30e-5p |  | hsa-miR-376a-3p |
| hsa-miR-376a-3p |  | hsa-miR-324-3p |  | hsa-miR-363-5p |  | hsa-miR-940 |  | hsa-miR-20a-3p |  | hsa-miR-31-5p |  | hsa-miR-376c-3p |
| hsa-miR-423-5p |  | hsa-miR-32-5p |  | hsa-miR-3652 |  | hsa-miR-942 |  | hsa-miR-21-3p |  | hsa-miR-320a |  | hsa-miR-377-3p |
| hsa-miR-424-5p |  | hsa-miR-331-3p |  | hsa-miR-3653 |  |  |  | hsa-miR-215 |  | hsa-miR-320b |  | hsa-miR-410 |
| hsa-miR-425-5p |  | hsa-miR-335-5p |  | hsa-miR-3663-3p |  |  |  | hsa-miR-21-5p |  | hsa-miR-320c |  | hsa-miR-4291 |
| hsa-miR-4291 |  | hsa-miR-33a-5p |  | hsa-miR-425-5p |  |  |  | hsa-miR-219-5p |  | hsa-miR-320d |  | hsa-miR-4313 |
| hsa-miR-4317 |  | hsa-miR-340-5p |  | hsa-miR-4286 |  |  |  | hsa-miR-221-5p |  | hsa-miR-320e |  | hsa-miR-431-5p |
| hsa-miR-4323 |  | hsa-miR-34a-5p |  | hsa-miR-4291 |  |  |  | hsa-miR-222-3p |  | hsa-miR-324-3p |  | hsa-miR-4317 |
| hsa-miR-4505 |  | hsa-miR-361-3p |  | hsa-miR-4317 |  |  |  | hsa-miR-223-3p |  | hsa-miR-32-5p |  | hsa-miR-4433-3p |
| hsa-miR-4507 |  | hsa-miR-362-3p |  | hsa-miR-4455 |  |  |  | hsa-miR-24-1-5p |  | hsa-miR-328 |  | hsa-miR-4507 |
| hsa-miR-4689 |  | hsa-miR-3656 |  | hsa-miR-4518 |  |  |  | hsa-miR-24-3p |  | hsa-miR-331-3p |  | hsa-miR-4532 |
| hsa-miR-4732-3p |  | hsa-miR-376a-3p |  | hsa-miR-4659a-3p |  |  |  | hsa-miR-25-5p |  | hsa-miR-338-3p |  | hsa-miR-4633-5p |
| hsa-miR-484 |  | hsa-miR-377-3p |  | hsa-miR-4707-3p |  |  |  | hsa-miR-26b-5p |  | hsa-miR-33a-5p |  | hsa-miR-4687-3p |
| hsa-miR-486-5p |  | hsa-miR-423-3p |  | hsa-miR-4732-5p |  |  |  | hsa-miR-27a-3p |  | hsa-miR-340-5p |  | hsa-miR-487b |
| hsa-miR-491-5p |  | hsa-miR-423-5p |  | hsa-miR-4739 |  |  |  | hsa-miR-27b-3p |  | hsa-miR-34a-5p |  | hsa-miR-493-5p |
| hsa-miR-500a-3p |  | hsa-miR-424-5p |  | hsa-miR-4763-3p |  |  |  | hsa-miR-28-5p |  | hsa-miR-361-3p |  | hsa-miR-495-3p |
| hsa-miR-501-3p |  | hsa-miR-4323 |  | hsa-miR-4800-5p |  |  |  | hsa-miR-29b-3p |  | hsa-miR-362-3p |  | hsa-miR-501-5p |
| hsa-miR-501-5p |  | hsa-miR-4484 |  | hsa-miR-484 |  |  |  | hsa-miR-29c-3p |  | hsa-miR-362-5p |  | hsa-miR-545-3p |
| hsa-miR-502-3p |  | hsa-miR-4505 |  | hsa-miR-486-3p |  |  |  | hsa-miR-301a-3p |  | hsa-miR-3663-3p |  | hsa-miR-548am-5p |
| hsa-miR-505-5p |  | hsa-miR-4507 |  | hsa-miR-486-5p |  |  |  | hsa-miR-301b |  | hsa-miR-423-3p |  | hsa-miR-551b-3p |
| hsa-miR-532-3p |  | hsa-miR-4513 |  | hsa-miR-491-5p |  |  |  | hsa-miR-30b-5p |  | hsa-miR-423-5p |  | hsa-miR-582-5p |
| hsa-miR-545-3p |  | hsa-miR-4633-5p |  | hsa-miR-5003-5p |  |  |  | hsa-miR-30d-5p |  | hsa-miR-424-5p |  | hsa-miR-598 |
| hsa-miR-548am-5p |  | hsa-miR-4651 |  | hsa-miR-500a-3p |  |  |  | hsa-miR-30e-3p |  | hsa-miR-425-5p |  | hsa-miR-6088 |
| hsa-miR-550a-3-5p |  | hsa-miR-4665-3p |  | hsa-miR-501-3p |  |  |  | hsa-miR-30e-5p |  | hsa-miR-4257 |  | hsa-miR-6124 |
| hsa-miR-550a-3p |  | hsa-miR-4689 |  | hsa-miR-502-3p |  |  |  | hsa-miR-3138 |  | hsa-miR-4270 |  | hsa-miR-624-5p |
| hsa-miR-551b-3p |  | hsa-miR-4690-5p |  | hsa-miR-505-5p |  |  |  | hsa-miR-31-5p |  | hsa-miR-4318 |  | hsa-miR-628-5p |
| hsa-miR-574-3p |  | hsa-miR-4732-3p |  | hsa-miR-532-3p |  |  |  | hsa-miR-3162-3p |  | hsa-miR-4323 |  | hsa-miR-654-3p |
| hsa-miR-582-5p |  | hsa-miR-4737 |  | hsa-miR-548q |  |  |  | hsa-miR-3195 |  | hsa-miR-4505 |  | hsa-miR-6723-5p |
| hsa-miR-590-5p |  | hsa-miR-484 |  | hsa-miR-550a-3-5p |  |  |  | hsa-miR-320a |  | hsa-miR-4507 |  | hsa-miR-6724-5p |
| hsa-miR-598 |  | hsa-miR-486-5p |  | hsa-miR-550a-3p |  |  |  | hsa-miR-320b |  | hsa-miR-450a-5p |  | hsa-miR-7-1-3p |
| hsa-miR-628-5p |  | hsa-miR-491-5p |  | hsa-miR-550a-5p |  |  |  | hsa-miR-320c |  | hsa-miR-4513 |  | hsa-miR-769-3p |
| hsa-miR-652-3p |  | hsa-miR-500a-3p |  | hsa-miR-550b-2-5p |  |  |  | hsa-miR-320d |  | hsa-miR-4659a-3p |  | hsa-miR-92a-3p |
| hsa-miR-7-1-3p |  | hsa-miR-501-3p |  | hsa-miR-582-5p |  |  |  | hsa-miR-320e |  | hsa-miR-4689 |  | hsa-miR-98-5p |
| hsa-miR-744-5p |  | hsa-miR-501-5p |  | hsa-miR-610 |  |  |  | hsa-miR-324-3p |  | hsa-miR-4707-3p |  | hsa-miR-99a-5p |
| hsa-miR-766-3p |  | hsa-miR-502-3p |  | hsa-miR-628-5p |  |  |  | hsa-miR-32-5p |  | hsa-miR-4732-3p |  |  |
| hsa-miR-92a-3p |  | hsa-miR-532-3p |  | hsa-miR-629-3p |  |  |  | hsa-miR-331-3p |  | hsa-miR-4732-5p |  |  |
|  |  | hsa-miR-542-3p |  | hsa-miR-641 |  |  |  | hsa-miR-335-5p |  | hsa-miR-484 |  |  |
|  |  | hsa-miR-545-3p |  | hsa-miR-652-3p |  |  |  | hsa-miR-338-3p |  | hsa-miR-486-5p |  |  |
|  |  | hsa-miR-548am-5p |  | hsa-miR-6723-5p |  |  |  | hsa-miR-33a-5p |  | hsa-miR-491-5p |  |  |
|  |  | hsa-miR-550a-3-5p |  | hsa-miR-7-1-3p |  |  |  | hsa-miR-340-5p |  | hsa-miR-500a-3p |  |  |
|  |  | hsa-miR-550a-3p |  | hsa-miR-92a-3p |  |  |  | hsa-miR-34a-5p |  | hsa-miR-5010-5p |  |  |
|  |  | hsa-miR-551b-3p |  |  |  |  |  | hsa-miR-361-3p |  | hsa-miR-501-3p |  |  |
|  |  | hsa-miR-574-3p |  |  |  |  |  | hsa-miR-362-3p |  | hsa-miR-501-5p |  |  |
|  |  | hsa-miR-582-5p |  |  |  |  |  | hsa-miR-3656 |  | hsa-miR-502-3p |  |  |
|  |  | hsa-miR-590-5p |  |  |  |  |  | hsa-miR-374a-5p |  | hsa-miR-505-5p |  |  |
|  |  | hsa-miR-598 |  |  |  |  |  | hsa-miR-376a-3p |  | hsa-miR-532-3p |  |  |
|  |  | hsa-miR-6073 |  |  |  |  |  | hsa-miR-376c-3p |  | hsa-miR-542-3p |  |  |
|  |  | hsa-miR-628-5p |  |  |  |  |  | hsa-miR-377-3p |  | hsa-miR-545-3p |  |  |
|  |  | hsa-miR-652-3p |  |  |  |  |  | hsa-miR-378g |  | hsa-miR-550a-3-5p |  |  |
|  |  | hsa-miR-652-5p |  |  |  |  |  | hsa-miR-3926 |  | hsa-miR-550a-3p |  |  |
|  |  | hsa-miR-7-1-3p |  |  |  |  |  | hsa-miR-423-3p |  | hsa-miR-550b-2-5p |  |  |
|  |  | hsa-miR-744-5p |  |  |  |  |  | hsa-miR-423-5p |  | hsa-miR-551b-3p |  |  |
|  |  | hsa-miR-766-3p |  |  |  |  |  | hsa-miR-424-5p |  | hsa-miR-574-3p |  |  |
|  |  | hsa-miR-770-5p |  |  |  |  |  | hsa-miR-4291 |  | hsa-miR-582-3p |  |  |
|  |  | hsa-miR-92a-3p |  |  |  |  |  | hsa-miR-4317 |  | hsa-miR-582-5p |  |  |
|  |  |  |  |  |  |  |  | hsa-miR-4323 |  | hsa-miR-590-5p |  |  |
|  |  |  |  |  |  |  |  | hsa-miR-4433-3p |  | hsa-miR-6073 |  |  |
|  |  |  |  |  |  |  |  | hsa-miR-4484 |  | hsa-miR-628-5p |  |  |
|  |  |  |  |  |  |  |  | hsa-miR-4505 |  | hsa-miR-629-3p |  |  |
|  |  |  |  |  |  |  |  | hsa-miR-4507 |  | hsa-miR-642a-3p |  |  |
|  |  |  |  |  |  |  |  | hsa-miR-450a-5p |  | hsa-miR-6508-5p |  |  |
|  |  |  |  |  |  |  |  | hsa-miR-4513 |  | hsa-miR-652-3p |  |  |
|  |  |  |  |  |  |  |  | hsa-miR-451a |  | hsa-miR-652-5p |  |  |
|  |  |  |  |  |  |  |  | hsa-miR-4532 |  | hsa-miR-744-5p |  |  |
|  |  |  |  |  |  |  |  | hsa-miR-454-5p |  | hsa-miR-766-3p |  |  |
|  |  |  |  |  |  |  |  | hsa-miR-4633-5p |  | hsa-miR-770-5p |  |  |
|  |  |  |  |  |  |  |  | hsa-miR-4651 |  | hsa-miR-92a-3p |  |  |
|  |  |  |  |  |  |  |  | hsa-miR-4665-3p |  | hsa-miR-93-3p |  |  |
|  |  |  |  |  |  |  |  | hsa-miR-4667-5p |  | hsa-miR-93-5p |  |  |
|  |  |  |  |  |  |  |  | hsa-miR-4689 |  | hsa-miR-942 |  |  |
|  |  |  |  |  |  |  |  | hsa-miR-4697-5p |  | hsa-miR-95 |  |  |
|  |  |  |  |  |  |  |  | hsa-miR-4737 |  |  |  |  |
|  |  |  |  |  |  |  |  | hsa-miR-484 |  |  |  |  |
|  |  |  |  |  |  |  |  | hsa-miR-486-5p |  |  |  |  |
|  |  |  |  |  |  |  |  | hsa-miR-491-5p |  |  |  |  |
|  |  |  |  |  |  |  |  | hsa-miR-495-3p |  |  |  |  |
|  |  |  |  |  |  |  |  | hsa-miR-497-5p |  |  |  |  |
|  |  |  |  |  |  |  |  | hsa-miR-500a-3p |  |  |  |  |
|  |  |  |  |  |  |  |  | hsa-miR-501-3p |  |  |  |  |
|  |  |  |  |  |  |  |  | hsa-miR-501-5p |  |  |  |  |
|  |  |  |  |  |  |  |  | hsa-miR-502-3p |  |  |  |  |
|  |  |  |  |  |  |  |  | hsa-miR-505-5p |  |  |  |  |
|  |  |  |  |  |  |  |  | hsa-miR-532-3p |  |  |  |  |
|  |  |  |  |  |  |  |  | hsa-miR-542-3p |  |  |  |  |
|  |  |  |  |  |  |  |  | hsa-miR-545-3p |  |  |  |  |
|  |  |  |  |  |  |  |  | hsa-miR-548am-5p |  |  |  |  |
|  |  |  |  |  |  |  |  | hsa-miR-550a-3-5p |  |  |  |  |
|  |  |  |  |  |  |  |  | hsa-miR-550a-3p |  |  |  |  |
|  |  |  |  |  |  |  |  | hsa-miR-551b-3p |  |  |  |  |
|  |  |  |  |  |  |  |  | hsa-miR-574-3p |  |  |  |  |
|  |  |  |  |  |  |  |  | hsa-miR-582-5p |  |  |  |  |
|  |  |  |  |  |  |  |  | hsa-miR-590-5p |  |  |  |  |
|  |  |  |  |  |  |  |  | hsa-miR-598 |  |  |  |  |
|  |  |  |  |  |  |  |  | hsa-miR-6127 |  |  |  |  |
|  |  |  |  |  |  |  |  | hsa-miR-628-5p |  |  |  |  |
|  |  |  |  |  |  |  |  | hsa-miR-6508-5p |  |  |  |  |
|  |  |  |  |  |  |  |  | hsa-miR-652-3p |  |  |  |  |
|  |  |  |  |  |  |  |  | hsa-miR-652-5p |  |  |  |  |
|  |  |  |  |  |  |  |  | hsa-miR-660-5p |  |  |  |  |
|  |  |  |  |  |  |  |  | hsa-miR-6724-5p |  |  |  |  |
|  |  |  |  |  |  |  |  | hsa-miR-7-1-3p |  |  |  |  |
|  |  |  |  |  |  |  |  | hsa-miR-744-5p |  |  |  |  |
|  |  |  |  |  |  |  |  | hsa-miR-766-3p |  |  |  |  |
|  |  |  |  |  |  |  |  | hsa-miR-874 |  |  |  |  |
|  |  |  |  |  |  |  |  | hsa-miR-92a-3p |  |  |  |  |

Supplementary Table S4: GO terms in the category of biological processes to be associated with the inversely correlated genes.

| **gene ontology term** | **term_goid** | **set size** | **contained** | **q-value** |
| --- | --- | --- | --- | --- |
| protein localization to organelle | GO:0033365 | 879 | 28 | 6.31E-08 |
| mRNA metabolic process | GO:0016071 | 652 | 20 | 3.20E-05 |
| regulation of mitotic cell cycle | GO:0007346 | 472 | 16 | 9.84E-05 |
| Wnt signaling pathway | GO:0016055 | 477 | 16 | 9.84E-05 |
| establishment of protein localization to endoplasmic reticulum | GO:0072599 | 105 | 8 | 0.000127 |
| RNA biosynthetic process | GO:0032774 | 3877 | 54 | 0.000194 |
| regulation of protein metabolic process | GO:0051246 | 2476 | 40 | 0.000194 |
| positive regulation of macromolecule metabolic process | GO:0010604 | 2795 | 43 | 0.000202 |
| negative regulation of cellular metabolic process | GO:0031324 | 2323 | 38 | 0.000202 |
| regulation of cellular protein metabolic process | GO:0032268 | 2327 | 38 | 0.000202 |
| RNA processing | GO:0006396 | 896 | 21 | 0.000203 |
| regulation of Wnt signaling pathway | GO:0030111 | 312 | 12 | 0.000203 |
| negative regulation of macromolecule metabolic process | GO:0010605 | 2297 | 37 | 0.00027 |
| positive regulation of cellular metabolic process | GO:0031325 | 2789 | 42 | 0.00027 |
| positive regulation of catabolic process | GO:0009896 | 328 | 12 | 0.00027 |
| protein complex assembly | GO:0006461 | 1281 | 25 | 0.000416 |
| RNA catabolic process | GO:0006401 | 242 | 10 | 0.000477 |
| muscle cell differentiation | GO:0042692 | 355 | 12 | 0.000491 |
| regulation of organ morphogenesis | GO:2000027 | 246 | 10 | 0.000491 |
| regulation of cellular protein localization | GO:1903827 | 547 | 15 | 0.000491 |
| negative regulation of cell differentiation | GO:0045596 | 624 | 16 | 0.000538 |
| regulation of cell development | GO:0060284 | 782 | 18 | 0.000676 |
| apoptotic process | GO:0006915 | 1817 | 30 | 0.000845 |
| translation | GO:0006412 | 657 | 16 | 0.000845 |
| angiotensin-activated signaling pathway involved in heart process | GO:0086098 | 2 | 2 | 0.00103 |
| regulation of morphogenesis of an epithelium | GO:1905330 | 175 | 8 | 0.00111 |
| negative regulation of nitrogen compound metabolic process | GO:0051172 | 1490 | 26 | 0.00113 |
| peptide biosynthetic process | GO:0043043 | 682 | 16 | 0.00113 |
| negative regulation of transferase activity | GO:0051348 | 349 | 11 | 0.00132 |
| regulation of cell cycle process | GO:0010564 | 557 | 14 | 0.00135 |
| regulation of epithelial cell proliferation | GO:0050678 | 293 | 10 | 0.00136 |
| positive regulation of transferase activity | GO:0051347 | 626 | 15 | 0.00141 |
| chondrocyte differentiation | GO:0002062 | 98 | 6 | 0.00141 |
| regulation of protein catabolic process | GO:0042176 | 364 | 11 | 0.00159 |
| regulation of cellular catabolic process | GO:0031329 | 368 | 11 | 0.00166 |
| regulation of gene expression | GO:0010468 | 4304 | 53 | 0.00166 |
| regulation of smooth muscle cell proliferation | GO:0048660 | 102 | 6 | 0.00166 |
| regulation of extent of cell growth | GO:0061387 | 103 | 6 | 0.00171 |
| positive regulation of cell growth | GO:0030307 | 149 | 7 | 0.00172 |
| regulation of neuron death | GO:1901214 | 255 | 9 | 0.00179 |
| proteasomal protein catabolic process | GO:0010498 | 377 | 11 | 0.00179 |
| positive regulation of cellular component organization | GO:0051130 | 1142 | 21 | 0.00193 |
| negative regulation of biosynthetic process | GO:0009890 | 1492 | 25 | 0.00193 |
| regulation of programmed cell death | GO:0043067 | 1409 | 24 | 0.00197 |
| regulation of cellular biosynthetic process | GO:0031326 | 4256 | 52 | 0.00201 |
| regulation of muscle cell differentiation | GO:0051147 | 157 | 7 | 0.00202 |
| regulation of macromolecule biosynthetic process | GO:0010556 | 4071 | 50 | 0.00241 |
| amide biosynthetic process | GO:0043604 | 765 | 16 | 0.00245 |
| positive regulation of nitrogen compound metabolic process | GO:0051173 | 1721 | 27 | 0.00245 |
| neuron apoptotic process | GO:0051402 | 216 | 8 | 0.00246 |
| axon extension | GO:0048675 | 118 | 6 | 0.00266 |
| regulation of glycoprotein metabolic process | GO:1903018 | 43 | 4 | 0.00279 |
| adherens junction assembly | GO:0034333 | 78 | 5 | 0.00284 |
| regulation of DNA metabolic process | GO:0051052 | 353 | 10 | 0.00348 |
| response to corticosteroid | GO:0031960 | 182 | 7 | 0.00425 |
| cellular protein catabolic process | GO:0044257 | 653 | 14 | 0.00425 |
| morphogenesis of a polarized epithelium | GO:0001738 | 132 | 6 | 0.00433 |
| regulation of organelle organization | GO:0033043 | 986 | 18 | 0.00455 |
| mitochondrial depolarization | GO:0051882 | 22 | 3 | 0.00459 |
| regulation of establishment of protein localization | GO:0070201 | 821 | 16 | 0.00459 |
| positive regulation of biosynthetic process | GO:0009891 | 1726 | 26 | 0.00478 |
| Ras protein signal transduction | GO:0007265 | 309 | 9 | 0.00478 |
| response to gamma radiation | GO:0010332 | 52 | 4 | 0.00478 |
| regulation of cell-matrix adhesion | GO:0001952 | 91 | 5 | 0.00478 |
| striated muscle cell development | GO:0055002 | 139 | 6 | 0.00499 |
| regulation of histone deacetylase activity | GO:1901725 | 6 | 2 | 0.00571 |
| regulation of nucleobase-containing compound metabolic process | GO:0019219 | 4066 | 48 | 0.00585 |
| cellular response to decreased oxygen levels | GO:0036294 | 146 | 6 | 0.00585 |
| transcription, DNA-templated | GO:0006351 | 3738 | 45 | 0.00585 |
| positive regulation of cell death | GO:0010942 | 612 | 13 | 0.00585 |
| negative regulation of cell communication | GO:0010648 | 1207 | 20 | 0.00612 |
| striated muscle hypertrophy | GO:0014897 | 59 | 4 | 0.00676 |
| cellular macromolecular complex assembly | GO:0034622 | 961 | 17 | 0.00683 |
| cellular protein modification process | GO:0006464 | 3772 | 45 | 0.00683 |
| regulation of monooxygenase activity | GO:0032768 | 60 | 4 | 0.00691 |
| negative regulation of cell death | GO:0060548 | 895 | 16 | 0.00874 |
| regulation of phosphorus metabolic process | GO:0051174 | 1632 | 24 | 0.00897 |
| phosphorylation | GO:0016310 | 2230 | 30 | 0.00897 |
| regulation of RNA metabolic process | GO:0051252 | 3727 | 44 | 0.00897 |
| regulation of cell projection organization | GO:0031344 | 575 | 12 | 0.00897 |
| cardiocyte differentiation | GO:0035051 | 112 | 5 | 0.00897 |
| collagen metabolic process | GO:0032963 | 111 | 5 | 0.00897 |
| regulation of type I interferon production | GO:0032479 | 112 | 5 | 0.00897 |
| response to X-ray | GO:0010165 | 32 | 3 | 0.00982 |
| positive regulation of cell differentiation | GO:0045597 | 830 | 15 | 0.00998 |
| gas homeostasis | GO:0033483 | 9 | 2 | 0.0104 |
| adherens junction organization | GO:0034332 | 116 | 5 | 0.0104 |
| negative regulation of signal transduction | GO:0009968 | 1102 | 18 | 0.0105 |
| G1/S transition of mitotic cell cycle | GO:0000082 | 232 | 7 | 0.0108 |
| regulation of intracellular signal transduction | GO:1902531 | 1683 | 24 | 0.0116 |
| regulation of cardiac muscle hypertrophy | GO:0010611 | 35 | 3 | 0.0116 |
| regulation of mesenchymal cell proliferation | GO:0010464 | 35 | 3 | 0.0116 |
| positive regulation of signal transduction | GO:0009967 | 1396 | 21 | 0.0116 |
| regulation of nervous system development | GO:0051960 | 767 | 14 | 0.0116 |
| ion homeostasis | GO:0050801 | 683 | 13 | 0.0116 |
| positive regulation of protein binding | GO:0032092 | 74 | 4 | 0.0117 |
| cellular response to organic cyclic compound | GO:0071407 | 527 | 11 | 0.0117 |
| nephron epithelium morphogenesis | GO:0072088 | 75 | 4 | 0.0121 |
| positive regulation of cytokine production | GO:0001819 | 386 | 9 | 0.0136 |
| establishment of protein localization to mitochondrion | GO:0072655 | 185 | 6 | 0.0138 |
| proteolysis involved in cellular protein catabolic process | GO:0051603 | 621 | 12 | 0.0138 |
| transmembrane receptor protein serine/threonine kinase signaling pathway | GO:0007178 | 316 | 8 | 0.0138 |
| cellular ion homeostasis | GO:0006873 | 547 | 11 | 0.015 |
| ERK1 and ERK2 cascade | GO:0070371 | 258 | 7 | 0.0163 |
| ncRNA metabolic process | GO:0034660 | 559 | 11 | 0.0167 |
| negative regulation of hydrolase activity | GO:0051346 | 405 | 9 | 0.0172 |
| cell-substrate junction assembly | GO:0007044 | 85 | 4 | 0.0173 |
| regulation of cell communication by electrical coupling | GO:0010649 | 13 | 2 | 0.0174 |
| Fc receptor mediated stimulatory signaling pathway | GO:0002431 | 138 | 5 | 0.0174 |
| negative regulation of cell proliferation | GO:0008285 | 653 | 12 | 0.0174 |
| positive regulation of ossification | GO:0045778 | 86 | 4 | 0.0174 |
| modification-dependent macromolecule catabolic process | GO:0043632 | 568 | 11 | 0.0181 |
| neuron projection extension involved in neuron projection guidance | GO:1902284 | 45 | 3 | 0.0191 |
| low-density lipoprotein receptor particle metabolic process | GO:0032799 | 14 | 2 | 0.0191 |
| positive regulation of cell size | GO:0045793 | 14 | 2 | 0.0191 |
| myeloid cell differentiation | GO:0030099 | 343 | 8 | 0.0191 |
| positive regulation of cellular component movement | GO:0051272 | 416 | 9 | 0.0191 |
| regulation of protein transport | GO:0051223 | 746 | 13 | 0.0195 |
| transmembrane receptor protein tyrosine kinase signaling pathway | GO:0007169 | 661 | 12 | 0.0195 |
| endochondral bone morphogenesis | GO:0060350 | 47 | 3 | 0.0205 |
| regulation of transmembrane receptor protein serine/threonine kinase signaling pathway | GO:0090092 | 209 | 6 | 0.0205 |
| osteoblast differentiation | GO:0001649 | 209 | 6 | 0.0205 |
| convergent extension | GO:0060026 | 15 | 2 | 0.0205 |
| regulation of cell-substrate junction assembly | GO:0090109 | 48 | 3 | 0.0209 |
| glycoprotein biosynthetic process | GO:0009101 | 352 | 8 | 0.0209 |
| telomere organization | GO:0032200 | 149 | 5 | 0.0209 |
| retina vasculature development in camera-type eye | GO:0061298 | 16 | 2 | 0.0226 |
| regulation of adherens junction organization | GO:1903391 | 50 | 3 | 0.0229 |
| ureteric bud development | GO:0001657 | 97 | 4 | 0.0229 |
| positive regulation of cell communication | GO:0010647 | 1525 | 21 | 0.0229 |
| regulation of innate immune response | GO:0045088 | 359 | 8 | 0.0229 |
| regulation of chromatin organization | GO:1902275 | 155 | 5 | 0.0233 |
| positive regulation of immune response | GO:0050778 | 687 | 12 | 0.0233 |
| positive regulation of protein complex assembly | GO:0031334 | 219 | 6 | 0.0233 |
| regulation of cell morphogenesis | GO:0022604 | 443 | 9 | 0.0236 |
| response to angiotensin | GO:1990776 | 17 | 2 | 0.0236 |
| camera-type eye development | GO:0043010 | 290 | 7 | 0.0236 |
| positive regulation of developmental growth | GO:0048639 | 158 | 5 | 0.0242 |
| cellular response to growth factor stimulus | GO:0071363 | 609 | 11 | 0.0242 |
| negative regulation of cell-substrate adhesion | GO:0010812 | 53 | 3 | 0.0246 |
| positive regulation of nervous system development | GO:0051962 | 447 | 9 | 0.0246 |
| cellular response to lipid | GO:0071396 | 528 | 10 | 0.0246 |
| macromolecule deacylation | GO:0098732 | 102 | 4 | 0.0246 |
| regulation of mitochondrial depolarization | GO:0051900 | 18 | 2 | 0.0251 |
| cellular response to peptide hormone stimulus | GO:0071375 | 300 | 7 | 0.0253 |
| regulation of kidney development | GO:0090183 | 55 | 3 | 0.0263 |
| negative regulation of sequestering of calcium ion | GO:0051283 | 106 | 4 | 0.0273 |
| axon development | GO:0061564 | 460 | 9 | 0.0283 |

Supplementary Table S5: GO terms in the category of biological processes to be associated with the positively correlated genes.

| **gene ontology term** | **term_goid** | **set size** | **contained** | **q-value** |
| --- | --- | --- | --- | --- |
| positive regulation of biosynthetic process | GO:0009891 | 1726 | 39 | 6.58E-05 |
| positive regulation of cellular metabolic process | GO:0031325 | 2789 | 52 | 8.96E-05 |
| positive regulation of macromolecule metabolic process | GO:0010604 | 2795 | 51 | 0.000157 |
| positive regulation of nitrogen compound metabolic process | GO:0051173 | 1721 | 36 | 0.000328 |
| regulation of macromolecule biosynthetic process | GO:0010556 | 4071 | 63 | 0.00101 |
| regulation of cellular biosynthetic process | GO:0031326 | 4256 | 64 | 0.00177 |
| negative regulation of nitrogen compound metabolic process | GO:0051172 | 1490 | 29 | 0.0075 |
| negative regulation of cellular metabolic process | GO:0031324 | 2323 | 39 | 0.00955 |
| androgen receptor signaling pathway | GO:0030521 | 61 | 5 | 0.00955 |
| regulation of gene expression | GO:0010468 | 4304 | 61 | 0.00955 |
| negative regulation of cell cycle | GO:0045786 | 513 | 14 | 0.00955 |
| negative regulation of biosynthetic process | GO:0009890 | 1492 | 28 | 0.00955 |
| G1/S transition of mitotic cell cycle | GO:0000082 | 232 | 9 | 0.00955 |
| regulation of nucleobase-containing compound metabolic process | GO:0019219 | 4066 | 58 | 0.00955 |
| regulation of RNA metabolic process | GO:0051252 | 3727 | 54 | 0.0106 |
| cellular response to steroid hormone stimulus | GO:0071383 | 242 | 9 | 0.011 |
| neuron differentiation | GO:0030182 | 1231 | 24 | 0.011 |
| DNA damage response, signal transduction by p53 class mediator | GO:0030330 | 108 | 6 | 0.011 |
| cellular protein modification process | GO:0006464 | 3772 | 54 | 0.011 |
| G1 DNA damage checkpoint | GO:0044783 | 73 | 5 | 0.011 |
| RNA biosynthetic process | GO:0032774 | 3877 | 55 | 0.011 |
| transcription, DNA-templated | GO:0006351 | 3738 | 53 | 0.014 |
| protein insertion into membrane | GO:0051205 | 48 | 4 | 0.0165 |
| protein localization to organelle | GO:0033365 | 879 | 18 | 0.024 |
| regulation of cellular protein metabolic process | GO:0032268 | 2327 | 36 | 0.024 |
| regulation of mitotic cell cycle | GO:0007346 | 472 | 12 | 0.024 |
| neurogenesis | GO:0022008 | 1433 | 25 | 0.026 |
| mitotic DNA damage checkpoint | GO:0044773 | 95 | 5 | 0.026 |
| stress-activated MAPK cascade | GO:0051403 | 246 | 8 | 0.029 |
| cellular response to decreased oxygen levels | GO:0036294 | 146 | 6 | 0.0301 |
| negative regulation of macromolecule metabolic process | GO:0010605 | 2297 | 35 | 0.0306 |
| cell morphogenesis involved in differentiation | GO:0000904 | 638 | 14 | 0.0326 |
| chromatin remodeling | GO:0006338 | 155 | 6 | 0.0381 |
| positive regulation of cell cycle | GO:0045787 | 328 | 9 | 0.0412 |
| cellular response to organic cyclic compound | GO:0071407 | 527 | 12 | 0.0426 |
| axon development | GO:0061564 | 460 | 11 | 0.0426 |
| cellular response to lipid | GO:0071396 | 528 | 12 | 0.0426 |
| regulation of protein metabolic process | GO:0051246 | 2476 | 36 | 0.0457 |
| dendritic spine morphogenesis | GO:0060997 | 38 | 3 | 0.0457 |
| apoptotic mitochondrial changes | GO:0008637 | 118 | 5 | 0.0457 |
| positive regulation of cell death | GO:0010942 | 612 | 13 | 0.0457 |
| embryonic epithelial tube formation | GO:0001838 | 119 | 5 | 0.0457 |
| apoptotic process | GO:0006915 | 1817 | 28 | 0.0484 |
| regulation of nervous system development | GO:0051960 | 767 | 15 | 0.0484 |
| negative regulation of cell differentiation | GO:0045596 | 624 | 13 | 0.0484 |
| response to UV | GO:0009411 | 128 | 5 | 0.0565 |
| regulation of cellular amide metabolic process | GO:0034248 | 363 | 9 | 0.058 |
| RNA catabolic process | GO:0006401 | 242 | 7 | 0.0592 |
| establishment of protein localization to mitochondrion | GO:0072655 | 185 | 6 | 0.0599 |
| intrinsic apoptotic signaling pathway in response to oxidative stress | GO:0008631 | 45 | 3 | 0.0605 |
| neuron projection extension involved in neuron projection guidance | GO:1902284 | 45 | 3 | 0.0605 |
| neural tube closure | GO:0001843 | 86 | 4 | 0.0605 |
| retina vasculature development in camera-type eye | GO:0061298 | 16 | 2 | 0.0611 |
| DNA damage response, signal transduction resulting in transcription | GO:0042772 | 16 | 2 | 0.0611 |
| DNA repair | GO:0006281 | 517 | 11 | 0.0611 |
| phosphorylation | GO:0016310 | 2230 | 32 | 0.0611 |
| endochondral bone morphogenesis | GO:0060350 | 47 | 3 | 0.0611 |

Supplementary Table S6: Overlap of the hallmark and oncogenic gene sets from the Molecular Signatures Database with the inversely correlated genes.

| **Gene Set Name [# Genes (K)]** | **Description** | **# Genes in Overlap (k)** | **k/K** | **p-value** | **FDR q-value** |
| --- | --- | --- | --- | --- | --- |
| HALLMARK_MYC_TARGETS_V1 [200] | A subgroup of genes regulated by MYC - version 1 (v1). | 14 |  | 8.52 e-15 | 2.04 e-12 |
| RAPA_EARLY_UP.V1_DN [191] | Genes down-regulated in BJAB (lymphoma) cells by rapamycin (sirolimus) [PubChem = 6610346]. | 8 |  | 2.99 e-7 | 3.57 e-5 |
| TBK1.DF_DN [287] | Genes down-regulated in epithelial lung cancer cell lines upon over-expression of an oncogenic form of KRAS [Gene ID=3845] gene and knockdown of TBK1 [Gene ID=29110] gene by RNAi. | 9 |  | 6.12 e-7 | 4.88 e-5 |
| ESC_V6.5_UP_EARLY.V1_DN [172] | Genes down-regulated during early stages of differentiation of embryoid bodies from V6.5 embryonic stem cells. | 7 |  | 2.03 e-6 | 1.22 e-4 |
| HALLMARK_IL2_STAT5_SIGNALING [200] | Genes up-regulated by STAT5 in response to IL2 stimulation. | 7 |  | 5.5 e-6 | 2.63 e-4 |
| HALLMARK_ANDROGEN_RESPONSE [101] | Genes defining response to androgens. | 5 |  | 2.42 e-5 | 9.64 e-4 |
| HALLMARK_UNFOLDED_PROTEIN_RESPONSE [113] | Genes up-regulated during unfolded protein response, a cellular stress response related to the endoplasmic reticulum. | 5 |  | 4.15 e-5 | 1.42 e-3 |
| ERB2_UP.V1_DN [197] | Genes down-regulated in MCF-7 cells (breast cancer) positive for ESR1 [Gene ID=2099] and engineered to express ligand-activatable ERBB2 [Gene ID=2064]. | 6 |  | 5.7 e-5 | 1.65 e-3 |
| HALLMARK_HYPOXIA [200] | Genes up-regulated in response to low oxygen levels (hypoxia). | 6 |  | 6.2 e-5 | 1.65 e-3 |
| CSR_EARLY_UP.V1_DN [154] | Genes down-regulated in early serum response of CRL 2091 cells (foreskin fibroblasts). | 5 |  | 1.79 e-4 | 4.28 e-3 |

Supplementary Table S7: Overlap of the hallmark and oncogenic gene sets from the Molecular Signatures Database with the positively correlated genes.

| **Gene Set Name [# Genes (K)]** | **Description** | **# Genes in Overlap (k)** | **k/K** | **p-value** | **FDR q-value** |
| --- | --- | --- | --- | --- | --- |
| HALLMARK_G2M_CHECKPOINT [200] | Genes involved in the G2/M checkpoint, as in progression through the cell division cycle. | 11 |  | 7.5 e-10 | 1.79 e-7 |
| HALLMARK_E2F_TARGETS [200] | Genes encoding cell cycle related targets of E2F transcription factors. | 8 |  | 1.83 e-6 | 2.19 e-4 |
| TBK1.DF_DN [287] | Genes down-regulated in epithelial lung cancer cell lines upon over-expression of an oncogenic form of KRAS [Gene ID=3845] gene and knockdown of TBK1 [Gene ID=29110] gene by RNAi. | 9 |  | 3.06 e-6 | 2.44 e-4 |
| HALLMARK_UNFOLDED_PROTEIN_RESPONSE [113] | Genes up-regulated during unfolded protein response, a cellular stress response related to the endoplasmic reticulum. | 6 |  | 7.38 e-6 | 4.41 e-4 |
| HALLMARK_IL2_STAT5_SIGNALING [200] | Genes up-regulated by STAT5 in response to IL2 stimulation. | 7 |  | 1.95 e-5 | 5.82 e-4 |
| HALLMARK_MITOTIC_SPINDLE [200] | Genes important for mitotic spindle assembly. | 7 |  | 1.95 e-5 | 5.82 e-4 |
| HALLMARK_MYC_TARGETS_V1 [200] | A subgroup of genes regulated by MYC - version 1 (v1). | 7 |  | 1.95 e-5 | 5.82 e-4 |
| HALLMARK_TNFA_SIGNALING_VIA_NFKB [200] | Genes regulated by NF-kB in response to TNF [GeneID=7124]. | 7 |  | 1.95 e-5 | 5.82 e-4 |
| HALLMARK_UV_RESPONSE_DN [144] | Genes down-regulated in response to ultraviolet (UV) radiation. | 6 |  | 2.93 e-5 | 7.78 e-4 |
| HALLMARK_MYC_TARGETS_V2 [58] | A subgroup of genes regulated by MYC - version 2 (v2). | 4 |  | 9.49 e-5 | 2.27 e-3 |
